# Activity dependent LoNA regulates translation by coordinating rRNA transcription and methylation

**DOI:** 10.1038/s41467-018-04072-4

**Published:** 2018-04-30

**Authors:** Dingfeng Li, Juan Zhang, Ming Wang, Xiaohui Li, Huarui Gong, Huiping Tang, Lin Chen, Lili Wan, Qiang Liu

**Affiliations:** 1 Hefei National Laboratory for Physical Sciences at the Microscale, School of Life Sciences, University of Science and Technology of China, Hefei, 230026 China; 2 CAS Key Laboratory of Brain Function and Disease, University of Science and Technology of China, Hefei, 230026 China; 3 Neurodegenerative Disease Research Center, University of Science and Technology of China, Hefei, 230026 China; 4 Department of Biochemistry and Biophysics, Howard Hughes Medical Institute, School of Medicine, University of Pennsylvania, Philadelphia, PA 19104 USA; 5 National Synchrotron Radiation Laboratory, University of Science and Technology of China, Hefei, 230029 China

## Abstract

The ribosome is indispensable for precisely controlling the capacity of protein synthesis. However, how translational machinery is coordinated to meet the translational demands remains elusive. Here, we identify a nucleolar-specific lncRNA (LoNA), its 5′ portion binds and sequesters nucleolin to suppress rRNA transcription, and its snoRNA like 3′ end recruits and diminishes fibrillarin activity to reduce rRNA methylation. Activity-dependent decrease of LoNA leads to elevated rRNA and ribosome levels, an increased proportion of polysomes, mRNA polysome loading, and protein translation. In addition, transport of ribosomes to synapses is particularly promoted, resulting in increased levels of AMPA/NMDA receptor, enhanced synaptic plasticity, long-term potentiation and consolidated memory. Strikingly, hippocampal LoNA deficiency not only enhances long-term memory in WT mice, but also restores impaired memory function in *APP/PS1* transgenic mice. Together, these findings reveal the multifaceted role of LoNA in modulating ribosome biogenesis to meet the translational demands of long-term memory.

## Abstract (web summary)

Non-coding RNAs have been shown to be key components in translational regulation. Here the authors characterize long nucleolus specific lncRNA termed LoNA, identified to play a role in protein biosynthesis by inhibiting rRNA production and ribosome biosynthesis in nucleoli.

## Introduction

Neuronal activities are known to regulate protein synthesis via multiple mechanisms, including phosphorylation of key transcription factors^1^, processing and maturation of both rRNAs and mRNAs^2,3^, and control of the nucleolar number^4^. Nucleolar numbers vary throughout development in neurons, suggesting that changes in the neuronal demands for protein synthesis are accommodated by regulation of nucleolar assembly^5^. rRNA, the RNA component of the ribosome, is essential for protein synthesis in all living organisms. The stimulation of neurons increases rRNA production^6^, and decreased rRNA synthesis and nucleolar disruption are primary signs of cellular stress associated with aging and neurodegenerative disorders. These observations suggest an essential role of nucleoli and ribosome RNA biogenesis implicated in learning and memory, as well as in neurological diseases.

Protein synthesis in neuronal cell bodies is undoubtedly important. However, local protein translation is proved to be crucial in synaptic development and plasticity^7–9^. A considerable number of mRNAs, including those encoding signaling molecules, scaffolds, and receptors, are transported to dendrites and synapses at appreciable levels^10^. Moreover, spine-localized polyribosomes are substantially increased upon potentiation^11^, indicating that there is delicate regulation of ribosome number/function to meet the demands of local protein synthesis. Evidence suggests that local translation is functionally indispensable for synaptic and behavioral plasticity^12^.

Non-coding RNAs (ncRNAs) are key regulators of translational control, and may regulate mRNAs via effects on protein translation, as well as by transcriptional and epigenetic mechanisms^13^. The local regulation of mRNA stability and translation is crucial for synaptic plasticity and is especially amenable to regulation by ncRNAs. For example, the brain cytoplasmic ncRNA *BC1/BC200* is associated with FMRP-mediated translational repression in dendrites^14^. However, these ncRNAs are localized to dendrites and synapses, and primarily function locally. Identification of ncRNAs in neuronal soma, the primary site of translational control, will further our understanding of translational control.

In the present study, we discover a long nucleolus-specific lncRNA (LoNA), that inhibits rRNA production and ribosome biosynthesis in nucleoli, and eventually protein synthesis, given its high expression level at resting state. Mechanistically, we show that the 5′ portion of LoNA harbors nucleolin (NCL)-binding sites and specifically binds to NCL, as the 3′ portion of LoNA contains an snoRNA that binds to fibrillarin (FBL). Using both in vitro and in vivo models, we demonstrate that LoNA reduces rRNA levels by attenuating the transcriptional activity of NCL, and also decreases rRNA 2′-*O*-methylation via diminishing FBL activity, together leading to suppressed rRNA production and altered ribosome heterogeneity. LoNA levels become substantially decreased in response to KCL application, repeated synaptic stimulation, or even behavioral training, which leads to the alleviation of rRNA synthesis suppression, consequently increased AMPA/NMDA receptor level, enhanced neuronal plasticity, improved LTP and long-term memory. Strikingly, LoNA knockdown is able not only to improve memory of WT mice, but also to functionally rescue the phenotype of impaired learning and memory of AD model mice. These findings reveal a nucleolar lncRNA that bridges rRNA transcription and post-transcriptional modification, suggesting a crucial role in translational control for long-lasting forms of synaptic plasticity and memory.

## Results

### Characterization of lncRNAs in nucleoli

**Fig. 1:**
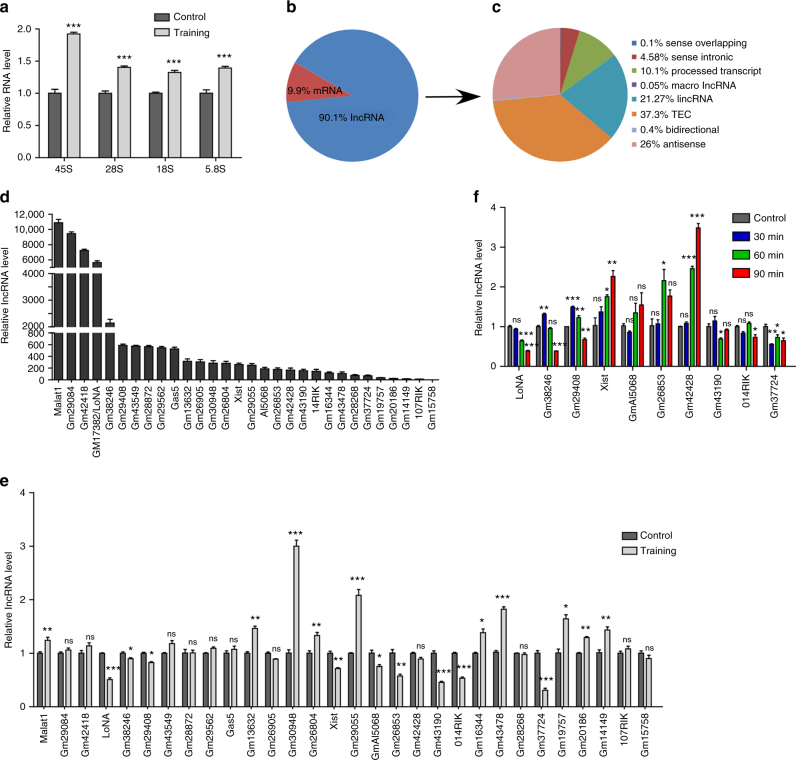
Identification of nucleolar lncRNAs from trained mouse brain and activated neurons. **a** rRNA levels (45S, 28S, 18S, and 5.8S) in hippocampal brain from spatial learning-induced mice in Morris water maze and control mice (24 h after training, *n* = 5 for each group), as measured by qPCR. Data were normalized against U1 snRNA. **b**, **c** Total RNA was extracted from N2a cell nucleoli, and fragments longer than 200 nt were subjected to RNA-seq. **b** Relative abundances of mRNA and lncRNA in nucleoli. **c** Distribution of detected nucleolar lncRNA RNA-seq peaks. **d** qPCR validation of expression for the 30 most abundant lncRNA in the nucleoli of N2a cells (*n* = 3), expression is presented as fold difference over Gm15758 (the least abundant one among the top thirty). **e** Expression of the 30 most abundant lncRNAs (validated in **d**) in hippocampal brain of experience induced and control mice (*n* = 5), as determined by qPCR. **f** Expression of the ten most significantly downregulated lncRNAs (determined in **e**) in KCL-activated primary neurons at various time point (*n* = 3 at each time point), determined by qPCR. In **a**,** d**, **e**, **f**, error bars, s.e.m.; *P < 0.05; **P < 0.01; ***P < 0.001 by ANOVA or two-tailed Student’s *t* test

Long-term memory is a fundamental cognitive process, requiring gene expression and protein synthesis for memory consolidation^15^. The biosynthesis of rRNA is essential in the production of functional ribosomes. To elucidate the role of rRNA in learning and memory, we have examined rRNA levels in mice with experience-induced memory formation using the Morris water maze and found that both pre-rRNA and mature rRNA levels were significantly elevated in trained mice as compared to controls (Fig. 1a and Supplementary Fig. 1a, b), indicating that rRNA transcription and/or processing play an essential role in activity-dependent learning and memory. Given that rRNA biosynthesis and processing takes place primarily at nucleoli, our goal was to characterize nucleolar lncRNAs and identify potential lncRNA regulators of rRNA production. Nucleolar RNA was isolated from N2a cells (Supplementary Fig. 1c), rRNA was removed and fragments longer than 200 nt were subjected to high-throughput RNA sequencing. Sequenced transcripts were then annotated using mouse genome mm10 and GENCODE (v19). For subsequent analyses, we considered two types of transcripts: protein-coding transcripts and long non-coding RNAs (lncRNAs). 9.9% of expressed transcripts were identified as mRNAs, with the remaining 90.1% annotated as lncRNAs (Fig. 1b, c), indicating that lncRNAs are far more abundant than protein-coding RNAs in nucleoli. Expression levels of the 30 most abundant lncRNAs were validated by qPCR (Fig. 1d). To further characterize the functions of these lncRNAs, we next measured their expression levels in hippocampal brain of mice subjected to Morris water maze training (Fig. 1e), as well as in activated cultured neurons (Fig. 1f and Supplementary Fig. 1d). We aimed to identify those lncRNAs whose levels were substantially reduced in trained mice and showed time-dependent decreases in activated cultured neurons. Using these criteria, we identified a lncRNA, GM17382, which is highly expressed in hippocampal brain (Supplementary Fig. 1e), and is particularly abundant in neurons (Supplementary Fig. 1f). In addition to the Morris water maze, GM17382 levels were also significantly reduced in mice subjected to fear conditioning and object-context discrimination training (Supplementary Fig. 1g, h, i, j).

### LoNA regulates rRNA transcription and protein translation

**Fig. 2:**
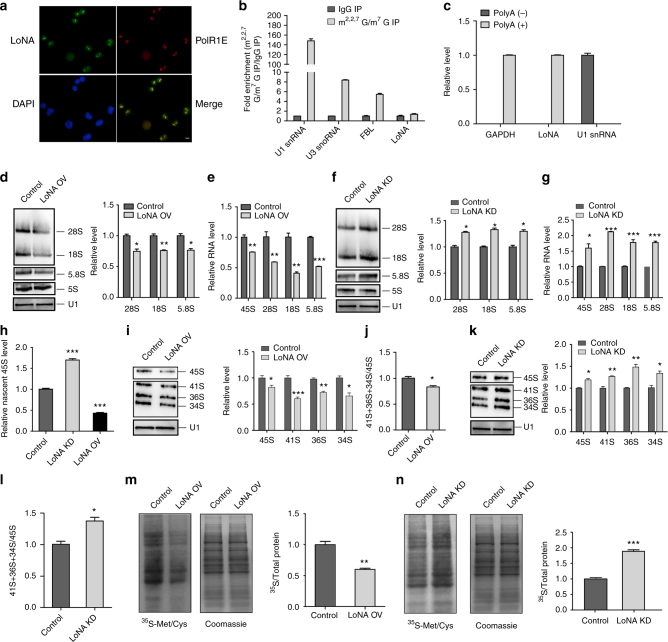
LoNA coordinates rRNA transcription and protein translation. **a** Nucleolar localization of LoNA. Representative immunofluorescence images of polR1E (red), LoNA RNA FISH (green), and DAPI (blue). Scale bar: 10 µm. **b** LoNA lacks m^2,2,7^G and m^7^G cap. IP was performed with total RNAs isolated from N2a cells using anti-m^2,2,7^G/m^7^G antibody or control IgG, followed by qPCR detection. Bar plots represent fold enrichments of immunoprecipitated RNAs by antibody over IgG control. U1 snRNA, U3 snoRNA, and FBL were included as positive controls. **c** LoNA is polyadenylated. poly (A)^+^ and poly (A)^−^ RNAs were purified from N2a total RNAs by oligo(dT) beads pulldown, followed by qPCR. Data were presented as relative abundance of a particular RNA in each pool. GAPDH was included as poly (A)^+^ control, U1 snRNA as poly (A)^−^ control. **d**–**g** Levels of mature rRNAs (28S, 18S, 5.8S, and 5S) were determined by Northern blot (U1 was used as control), densitometric analysis, and qPCR (data were normalized to U1 snRNA) in LoNA-overexpressed N2a cells (LoNA OV) (**d**–**e**), or LoNA knockdown N2a cells (LoNA KD) (**f**, **g**). **h** Nascent pre-rRNA (45S) levels were determined by nuclear run-on assay. **i**–**l** Levels of pre-rRNA (45S) and intermediate rRNAs (41S, 36S, and 34S) were determined in LoNA transfected N2a cells (LoNA OV) by Northern blot (U1 was used as control), densitometric analysis (**i**) and qPCR (45S only) (**e**), as well as in LoNA-deficient N2a cells (LoNA KD) by Northern blot (U1 was used as control), densitometric analysis (**k**) and qPCR (45S only) (**g**). **j**, **l** Ratio of total intermediate rRNA to the precursor 45S, as determined by densitometric analysis. **m**, **n** LoNA administered or deficient N2a cells were pulsed with ^35^S-Met/Cys, de novo protein synthesis was quantified as the mean ratio of ^35^S incorporation relative to total protein (Coomassie). For this and subsequent figures, OV represents overexpression, KD represents knockdown. Sequences of Northern blot probes and qPCR primers are listed in supplementary materials. *P < 0.05; **P < 0.01; ***P < 0.001 by ANOVA or two-tailed Student’s *t* test, error bars, s.e.m.

Imaging by immunostaining and FISH revealed that GM17382 co-localizes with polR1E (Fig. 2a), indicating that it is enriched specifically in nucleoli. We therefore renamed this long nucleolar ncRNA to LoNA. Nucleolar localization suggests that LoNA is not prone to translation. LoNA contains two exons (0.2 and 1.3 kb in length, respectively) and one intron (25 kb), but no obvious open reading frame. Its mature form is 1.5 kb long without any other detectable band by Northern blot (Supplementary Fig. 2a), indicating that no shorter form was produced. Interestingly, immunoprecipitation (IP) showed that LoNA is neither 2,2,7-trimethyl guanosine (m^2,2,7^G) nor 7-monomethyl guanosine (m^7^G) capped at the 5′ end (Fig. 2b), but is polyadenylated at the 3′ end as indicated by oligo (dT) RIP (Fig. 2c). We next investigated which RNA polymerase is responsible for the transcription of LoNA. Treatment with various polymerase inhibitors indicated that LoNA transcription is dependent on polII, but not polI or polIII (Supplementary Fig. 2b, c, d). Given the specific localization of LoNA to nucleoli, we next explored whether LoNA alters rRNA levels. N2a cells were transfected with either LoNA directly to simulate overexpression (Supplementary Fig. 2e), or with LoNA shRNA (Supplementary Fig. 2f) or antisense oligo (ASO) (Supplementary Fig. 2g) to suppress LoNA expression. In addition to qPCR, LoNA expression levels were verified by Northern blot (Supplementary Fig. 2h). Northern blot analysis of mature rRNA revealed that the levels of 28S, 18S, and 5.8S rRNAs were markedly decreased in response to administration of LoNA (Fig. 2d) and increased when LoNA was suppressed (Fig. 2f). However, expression of 5S rRNA, whose transcription is polIII dependent, was not impacted (Fig. 2d, f). These observations were also validated by qPCR (Fig. 2e, g and Supplementary Fig. 2i). Importantly, these results were reproducible in LoNA-deficient primary neuronal cells (Supplementary Fig. 2j, k). We next investigated whether these changes in rRNA expression resulted from altered rRNA transcription or from subsequent processing. Northern blot analysis showed that the level of 45S rRNA precursors was substantially decreased in the presence of LoNA (Fig. 2i) and increased in the absence of LoNA (Fig. 2k). qPCR-based measurements support these observations (Fig. 2e, g and Supplementary Fig. 2i, k). In addition, nuclear run-on analysis demonstrated that nascent 45S levels were considerably downregulated in LoNA-overexpressed cells and upregulated in LoNA-deficient cells (Fig. 2h). These observations indicate that LoNA regulates rRNAs at the transcriptional level. Moreover, levels of some of the intermediate rRNAs, including 41S, 36S, and 34S, were also altered in response to LoNA overexpression or suppression, as measured by Northern blot (Fig. 2i, k). In addition, densitometric analyses suggested that the ratio of total intermediate rRNA to pre-rRNA 45S was slightly altered by LoNA (Fig. 2j, l), suggesting that rRNA processing was marginally affected. We also calculated the copy number (CN) of LoNA to assess its sufficiency to inhibit rDNA transcription, and found that the CN of LoNA in N2a cells and primary neurons were approximately 739 and 1046 copies per cell, respectively (Supplementary Fig. 2l). By comparison, Gibbons^16^ et al. estimated that the CN of rDNA in the mouse genome ranges from 31 to 289. This finding indicates that LoNA is far more abundant than rDNA and is capable of modulating rDNA transcription. To assess whether LoNA influences translation, we pulsed N2a cells transfected with LoNA or control plasmid with ^35^S-Met/Cys as a measurement of de novo protein synthesis, and discovered that LoNA-overexpressed N2a cells displayed significantly decreased protein synthesis rates (Fig. 2m), whereas LoNA knockdown cells displayed increased protein synthesis rates (Fig. 2n). The effect of LoNA on protein synthesis was also evident in LoNA-deficient primary neuronal cells (Supplementary Fig. 2m). Taken together, these results indicate that LoNA inhibits rRNA transcription, and subsequent protein translation.

### LoNA modulates rRNA transcription by interaction with NCL

**Fig. 3:**
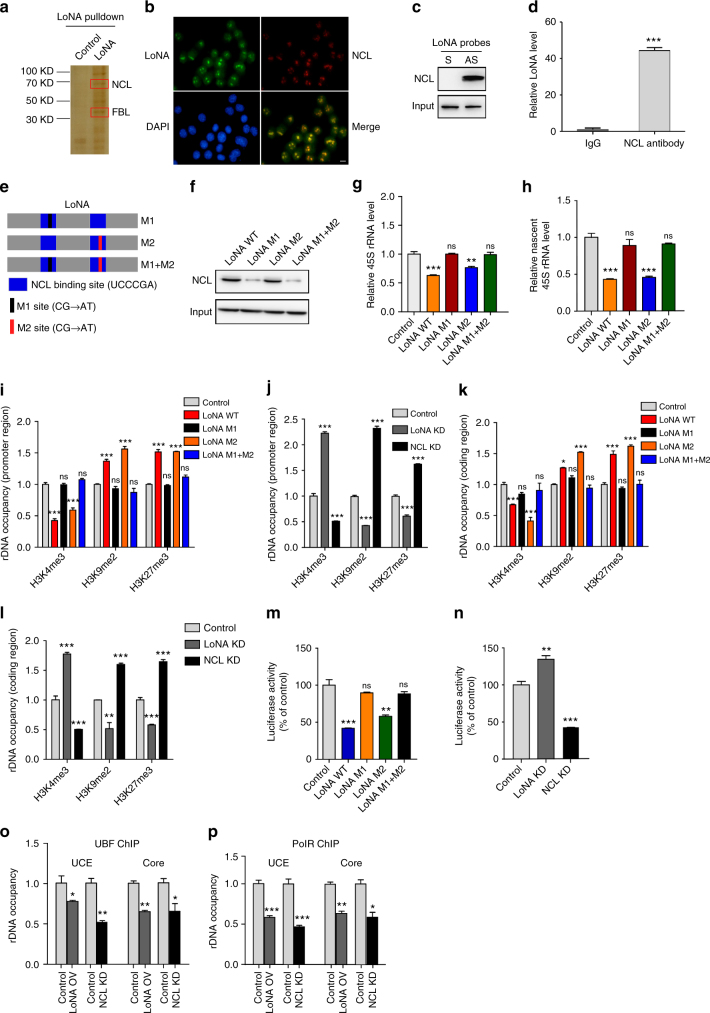
LoNA modulates rRNA transcription by interacting with NCL. **a** Silver staining of proteins pulled down with biotinylated LoNA antisense DNA probe or control probe with sense DNA sequence. Red square denotes the bands identified as NCL and FBL by mass spectrometry. **b**–**d** Verification of LoNA and NCL association. **b** Representative immunofluorescence images of NCL (red), LoNA RNA FISH (green), and DAPI (blue). Scale bar: 10 µm. **c** NCL was pulled down by LoNA DNA antisense probes by RIP, level was determined by western blot. Total NCL level was determined as input. **d** LoNA was pulled down by anti-NCL antibody, level was determined by qPCR. **e** Schematic of WT LoNA and three LoNA mutant variants (M1, M2, M1+M2). **f** Mutant LoNA showed diminished binding affinity to NCL. Binding affinity of NCL to WT or mutant LoNA were determined by pulldown of biotinylated full-length RNA. Total NCL level was used as input control. **g** Pre-rRNA 45S levels were determined by qPCR in the presence of WT or mutant LoNAs, 45S level was normalized to U1. **h** Nascent 45 S rRNA levels were measured by nuclear run-on analysis in the presence of WT or mutant LoNAs, 45S level was normalized to U1. **i**–**l** LoNA alters histone methylation states in the rDNA promoter (**i**, **j**) and coding regions (**k**,** l**) in N2a cells, as determined by ChIP with H3K4me3, H3K9me2, or H3K27me3 antibody, respectively. NCL knockdown N2a cells were included as positive controls. **m**–**n** 45S rRNA promoter in a luciferase vector was introduced to N2a cells containing WT LoNA, mutant LoNAs, LoNA shRNA, or NCL shRNA, respectively. Promoter activity was determined by luciferase assay. NCL knockdown N2a cells were included as positive controls. **o** LoNA administered N2a cells showed decreased UBF loading on UCE and core element of rDNA promoter region as determined by UBF ChIP. **p** LoNA administrated N2a cells exhibited decreased polI loading on UCE, core element, as determined by polI ChIP. NCL knockdown N2a cells were included as positive controls. *P < 0.05; **P < 0.01; ***P < 0.001 by ANOVA or two-tailed Student’s *t* test, error bars, s.e.m.

To further explore the role of LoNA in rRNA transcription and protein translation, we generated multiple antisense biotin-labeled fragments that span nearly the entire length of LoNA, along with sense biotin-labeled fragments as controls, and performed RNA pulldown experiments to identify LoNA-binding proteins by SDS-PAGE and subsequent mass spectrometry (MS). We first validated this method by quantifying levels of probe pulldown LoNA (Supplementary Fig. 3a). These assays revealed that LoNA associates with nucleolin (NCL) (Fig. 3a), which has been shown to alter the epigenetic state of rDNA and therefore to modulate the biosynthesis of rRNAs^17,18^. FISH and immunostaining confirmed that NCL co-localizes with LoNA in cells (Fig. 3b). The association between LoNA and NCL was further validated by western blot of proteins pulled down by LoNA antisense or control DNA probes, and then immunoblotted with anti-NCL antibody (Fig. 3c), and by qPCR analysis of RNAs pulled down by anti-NCL antibody (Fig. 3d and Supplementary Fig. 3b, c). In addition, NCL binding of LoNA exhibited a dose-dependent increase in response to elevated LoNA levels (Supplementary Fig. 3d). Since LoNA contains two UCCCGA NCL-binding motifs^19^, we next generated LoNA mutants targeting one or both of these motifs (M1, M2, and M1 + M2) (Fig. 3e). To evaluate whether LoNA binds to NCL through these binding motifs, we performed pulldown of biotinylated RNA and found that LoNA sequences harboring mutations M1 or M1 + M2 exhibited substantially weaker binding affinity to NCL compared to WT LoNA, whereas LoNA harboring mutations M2 had similar binding affinity to WT (Fig. 3f). UV-CLIP assay further confirmed the specific interactions of LoNA and NCL (Supplementary Fig. 3e). These results indicate that LoNA binds to NCL primarily through the M1 binding site. We next transfected N2a cells with WT, M1, M2, or M1 + M2 LoNA to investigate whether these variants alter rRNA transcription (Supplementary Fig. 3f). N2a cells transfected with WT or M2 mutant LoNA showed reduced expression of 45S rRNA, whereas M1 or M1 + M2 LoNA mutants were comparable to vector controls (Fig. 3g). Moreover, newly synthesized 45S exhibited similar changes as steady state of 45S in response to WT and mutant LoNA (Fig. 3h). NCL-deficient cells were included as a positive control (Supplementary Fig. 3g, h, i). However, the distribution of NCL was not influenced by LoNA level (Supplementary Fig. 3j), and the expression of NCL was not affected by WT LoNA or any of the LoNA mutants (Supplementary Fig. 3k, l, m). These observations suggest that LoNA binds directly to NCL primarily through the M1 binding site and sequesters NCL activity in rRNA biosynthesis. Histone methylation is known to be crucial to the establishment of active and inactive rDNA chromatins^20,21^, we therefore examined histone epigenetic states. LoNA-overexpressed N2a cells showed decreased levels of histone H3 tri-methylation on lysine 4 (H3K4me3, a marker of active chromatin), increased levels of H3 di-methylation on lysine 9 and H3 tri-methylation on lysine 27 (H3K9me2, H3K27me3, both markers of repressed chromatin) in rDNA promoters (Fig. 3i) and coding regions (Fig. 3k). This result suggests that LoNA leads to an inactive rDNA chromatin state. Overexpression of M1 or M1 + M2 LoNA mutants did not recapitulate the effects of WT LoNA on chromatin state, whereas the M2 LoNA mutant impacted chromatin state in a manner comparable to WT LoNA (Fig. 3i, k). LoNA knockdown cells also supported these notions, and NCL-deficient N2a cells were included as positive controls (Fig. 3j, l). To evaluate if LoNA regulates rDNA promoter activity, a luciferase reporter construct containing a 2kb segment of rDNA promoter region was transfected into N2a cells with LoNA or mutant LoNA. Luciferase activity indicated that LoNA, but not the LoNA M1 mutant, suppressed rDNA promoter activity (Fig. 3m). On the other hand, the promoter activity was increased in LoNA-deficient cells, and NCL knockdown cells were included as a positive control (Fig. 3n). The biosynthesis of 45S rRNA is PolI-dependent and upstream binding factor (UBF) is its specific transcription initiation factor^22^. We next investigated UBF loading on rRNA chromatins. N2a cells were transfected with LoNA and subjected to chromatin IP (ChIP). UBF ChIP indicated that transfection with LoNA significantly decreased UBF loading on the upstream control element (UCE) and core element on the rDNA promoter region (Fig. 3o), but did not alter total UBF level, as measured by western blot (Supplementary Fig. 3m). In addition, LoNA overexpression reduced RNA polymerase (Pol) I loading on the UCE, core element of rRNA chromatin (Fig. 3p). Similar changes were observed in NCL-deficient cells (Fig. 3o, p). Together, these observations indicate that increased LoNA leads to an inactive chromatin state in rDNA promoter and coding regions, as well as decreased UBF and PolI loading on UCE and core promoter regions. These findings also suggest that the impact of LoNA on chromatin state is achieved by binding to NCL to diminish its activity, rather than its expression.

### LoNA binds to FBL and alters rRNAs methylation status

**Fig. 4:**
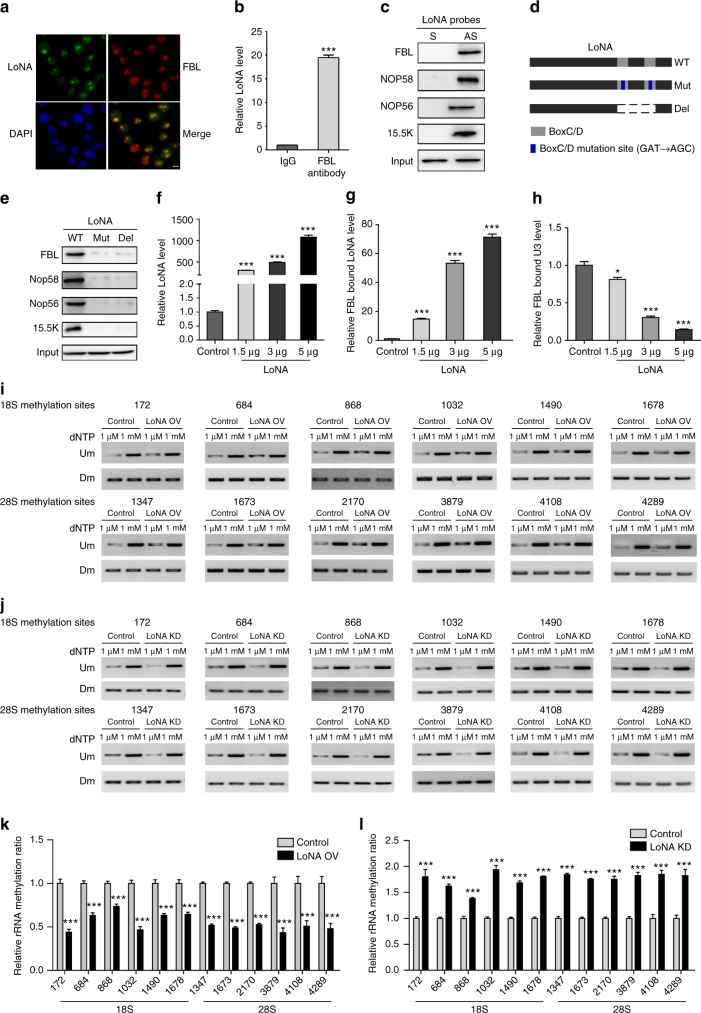
LoNA binds to FBL and attenuates its function on rRNAs methylation. **a**–**c** FBL is in association with LoNA. **a** Representative immunofluorescence images of FBL (red), LoNA RNA FISH (green), and DAPI (blue) show co-localization of LoNA and FBL. Scale bar: 10 µm. **b** LoNA was pulled down by anti-FBL antibody, and its level was determined by qPCR. **c** FBL, NOP58, NOP56, and 15.5k protein were concomitantly pulled down by LoNA DNA antisense probes, proteins levels were determined by western blot. GAPDH was included as an input control. **d** Schematic of WT, box C/D mutated (Mut), and box C/D deleted (Del) LoNA. **e** The box C/D structure of LoNA is essential for binding to FBL, NOP56, NOP58, and 15.5k. Binding affinity of FBL/NOP56/NOP58/15.5k to WT, Mut, or Del LoNAs was determined by pulldown of biotinylated full-length RNA. GAPDH was included as an input control. **f** Relative levels of LoNA detected in N2a cells transfected with increasing amounts of WT LoNA. **g** In response to increasing levels of LoNA, level of FBL-bound LoNA was increased, **h** whereas FBL-bound U3 snoRNA was decreased, both in a dose-dependent manner, as measured by IP with FBL antibody, followed by qPCR. **i**-**j** The detection of 12 methylation sites distributed throughout the 18S and 28S rRNA by RTL-P. Six sites were determined for the 18S, and other six for the 28S. Total RNAs from LoNA-overexpressed (**i**) or knockdown N2a cells (**j**) were subjected to RT with RT primer at low (1 µM) or high (1 mM) concentration of dNTP, respectively. cDNA was then amplified with primer pairs corresponding to upstream (Um) or downstream (Dm) regions of a specific methylation site. **k**, **l** Densitometric analysis of **i**, **j**, data were presented as signal intensity ratio of amplification products at low dNTP (1 µM) over high dNTP (1 mM) level (*n* = 3). Methylation levels in LoNA-overexpressed N2a cells (normalized to control N2a cells) (**k**), and in LoNA-deficient N2a cells (normalized to control N2a cells) (**l**). The position of the analyzed nucleotide was indicated in *x*-axis. Error bars, s.e.m.; *P < 0.05; **P < 0.01; ***P < 0.001 by ANOVA or two-tailed Student’s *t* test

MS analysis also identified fibrillarin (FBL) as a putative LoNA-binding protein. In eukaryotes, FBL is the only known methyltransferase that performs specific 2′-*O*-ribose-methylation, guided by snoRNAs such as U3. snoRNAs are active as part of small nucleolar ribonucleoprotein particle complexes (snoRNPs), with Box C (RUGAUAG; where R designates a purine) and Box D (CUGA) forming a functional structure^23^ to recruit FBL and facilitate rRNA methylation^24,25^. Given that LoNA harbors two box C/D sequences, we next examined whether LoNA binds to FBL through box C/D to modulate rRNAs post-transcriptionally. FISH and immunostaining indicated that LoNA co-localized with FBL (Fig. 4a). RNA immunoprecipitation and RNA pulldown further demonstrated that FBL was associated with LoNA, also indicated that LoNA associates with NOP58, NOP56, and 15.5k (NHPX) protein (Fig. 4b, c and Supplementary Fig. 4a). These observations suggest that LoNA interacts with snoRNPs complexes. We next generated a LoNA mutant construct with two mutations on each of its box C/D regions (Mut), and a deletion mutant construct with both box C/D sequences removed (Del), as indicated in Fig. 4d. Biotinylated Mut or Del LoNA pulldown assays revealed that both Mut and Del LoNA variants exhibited extremely weak binding affinities not only to FBL, but also to other components of snoRNP complex, as compared to WT LoNA (Fig. 4e), indicating that LoNA binds to FBL primarily through its box C/D structures. UV-CLIP assays also supported the specific interactions of LoNA and FBL (Supplementary Fig. 4b). Biotinylated M1, M2, or M1 + M2 LoNA (mutations on LoNA NCL-binding sites) showed no preferential binding affinity to FBL as compared to WT LoNA (Supplementary Fig. 4c). Similarly, biotinylated Mut or Del LoNA exhibited similar binding affinity to NCL as WT LoNA (Supplementary Fig. 4d), indicating that binding of LoNA to NCL and FBL are independent. We also observed that FBL-bound LoNA is increased in a dose-dependent manner with respect to the level of LoNA, while FBL-bound U3 was reduced by LoNA in a dose-dependent manner (Fig. 4f–h). Moreover, the distribution of FBL was not influenced by LoNA expression (Supplementary Fig. 4h), and the level of FBL was not affected by WT, Mut, or Del LoNA (Supplementary Fig. 4e, f, g). These observations suggest that LoNA binds to FBL competitively with U3 through its box C/D sequences, reducing FBL’s association with snoRNAs but not its expression nor distribution, and possibly impairs snoRNP function. Since snoRNP-mediated rRNA methylation represents the most prominent source of ribosome heterogeneity^26,27^, we next assessed the heterogeneity of rRNA methylation at 12 sites distributed along the 18S and 28S rRNA that were previously reported to be methylated^28^. N2a cells were transfected with WT, Mut, or Del LoNA for overexpression (Supplementary Fig. 4i), or with shRNA for knockdown. The determination of methylation levels of 12 methylation sites distributed throughout the 18S and 28S rRNA were conducted by RTL-P, (details were noted in Supplementary Fig. 5). The methylation levels across these 12 sites were significantly decreased in the presence of WT LoNA (Fig. 4i, k) and increased in the absence of WT LoNA (Fig. 4j, l). Importantly, administration of LoNA Mut or Del had no effect on rRNA methylation (Supplementary Fig. 6a, b, d, e), indicating that rRNA methylation status was regulated by LoNA through its box C/D structures, and suggesting that snoRNP function was modulated accordingly. rRNA methylation levels in N2a cells with FBL knockdown (a positive control) were phenotypically similar to N2a cells with LoNA overexpression (Supplementary Fig. 4j, 6c, f).

### LoNA alters polysome distribution and synaptic plasticity

**Fig. 5:**
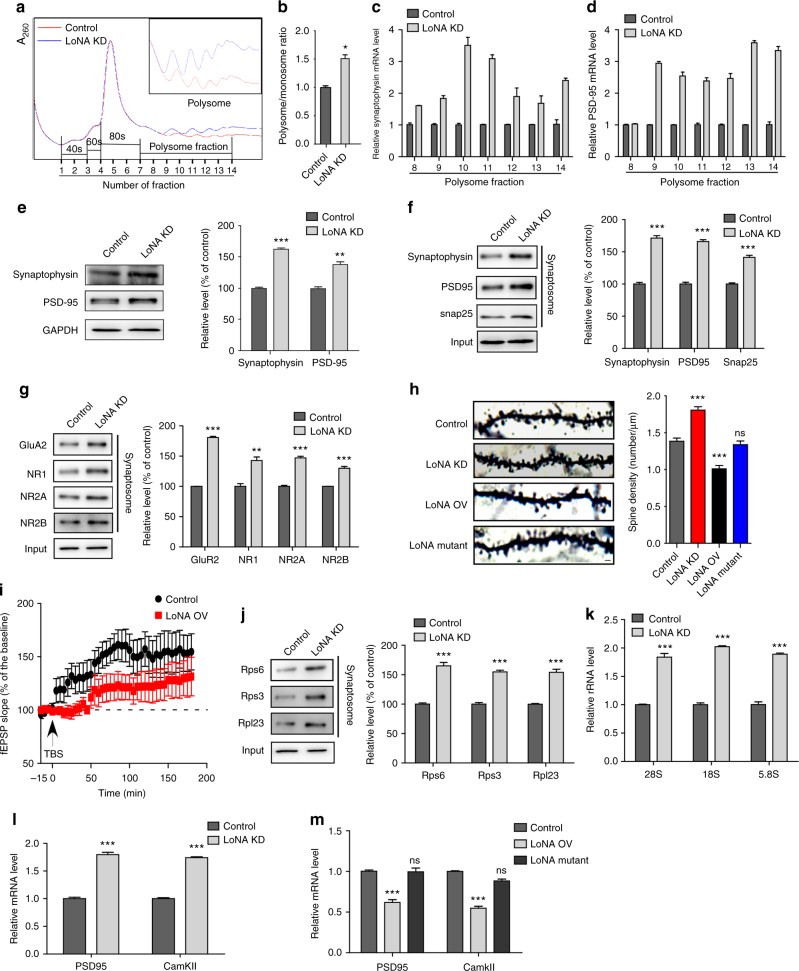
LoNA alters polysome distribution, synaptic plasticity, and LTP. **a** LoNA alters polysome distributions. Cytoplasmic polysome patterns of control (red) and LoNA-deficient (blue) N2a cells were denoted. **b** Quantification of the ratio of polysomes to the monosomes (80S) (mean ± s.d., *n* = 3). **c**, **d** Polysomal-bound mRNA levels of synaptophysin (**c**) and PSD95 (**d**) were determined in polysome fractions of LoNA deficient and control N2a cells by qPCR. Bar plots represent fold changes in LoNA deficient over control N2a cells (*n* = 3). **e** LoNA-deficient N2a cells were pulsed with puromycin, followed by immunoprecipitation with synaptophysin or PSD95 antibodies and immunoblotting with puromycin antibody. GAPDH was used as an input control. **f**,** g** Synaptosome fractions were purified from equal amounts of hippocampus from LoNA deficient and control mice. **f** Levels of synaptic protein synaptophysin, PSD95, and snap25 were determined by western blot and densitometric analysis (*n* = 5). **g** Level of AMPA receptor GluA2, NMDA receptor NR1, NR2A, and NR2B were measured by western blot and densitometric analysis (*n* = 5). GAPDH was used as an input control. **h** Representative images of Golgi-impregnated pyramidal neurons in layers II/III of the CA1 region of the hippocampus from LoNA deficient, LoNA and mutant LoNA administered mice. Spine density was quantified along dendrites among these mice (*n* = 5). Scale bar: 1 µm. **i** LTP deficits in LoNA-administered mice. The theta burst stimulus used to evoke CA1 LTP in control and LoNA-administered mice consisted of five trains of 20 pulses at 200 Hz stimulation for 1 s with each train separated by a 1 min interval (*n* = 10 for each group). **j**, **k** Synaptosome fractions were purified from equal amounts of hippocampus from LoNA deficient and control mice. **j** Levels of ribosomal protein Rps6, Rps3, and Rpl23 were measured by western blot and densitometric analysis (*n* = 5). **k** rRNA levels were determined by qPCR (*n* = 5). **l**, **m** Ribosomes were further purified from synaptosome fractions, and ribosome-bound mRNAs were measured by qPCR in LoNA deficient mice (**l**) or LoNA-overexpressed mice (**m**) (*n* = 5, each group). Error bars, s.e.m.; *P < 0.05; **P < 0.01; ***P < 0.001 by ANOVA or two-tailed Student’s *t* test

Fractionation of polysomes allows examination of mRNA bound to more than one ribosome and, therefore, contains mRNAs engaging in active translation. We performed polysome profiling assays to monitor cytoplasmic translation, and found that LoNA-depleted N2a cells exhibited an increased proportion of polysomes (Fig. 5a), suggesting that LoNA decreases translational efficiency. Averaged readings for each polysomal fraction from three independent profiling experiments supported this conclusion (Supplementary Fig. 7a), as did calculation of the ratio of polysomes to monosomes (Fig. 5b). Deficiency of LoNA did not affect the 80S peak, but did enhance the polysome:monosome ratio. This finding could indicate that the respective ribosomes are prior to engaging in translation. Ribosomal protein Rps6 is used as an identifier for the small ribosomal subunit and indicates that polysome profiling was successful (Supplementary Fig. 7b). We next examined the level of ribosome-bound synaptophysin and postsynaptic density-95 (PSD95) mRNA, which are presynaptic and postsynaptic markers, respectively, and observed a significant increase of both in polysomal fractions from LoNA-knockdown N2a cells (Fig. 5c, d). Consistent with these observations, we also found a substantial increase of newly synthesized synaptophysin and PSD95 proteins using LoNA-deficient N2a cells pre-fed with puromycin (i.e., SUnSET assay), as well as by densitometric analyses (Fig. 5e). To further assess the role of LoNA in synapse formation and plasticity in vivo, we knocked down or overexpressed LoNA, including mutant LoNA that simultaneously harbors both M1 mutant and box C/D Del, specifically in the hippocampal brain using adeno-associated virus (AAV) delivery technology (Supplementary Fig. 7c, d). TUNEL staining on injected brain sections indicated that adeno virus itself did not induce cell death in brains (Supplementary Fig. 7e). Synaptosomes were purified from hippocampal brain of these mice and levels of the synaptic proteins, as well as AMPA and NMDA receptors were determined by western blot. Our results showed that the levels of the synaptic proteins synaptophysin, snap25, PSD95, AMPA receptor GluA2, NMDA receptor NR1, NR2A and NR2B were significantly elevated in synaptosome fraction isolated from LoNA knockdown mice (Fig. 5f, g), reduced in LoNA-overexpressed mice and unaltered in mutant LoNA-recipient mice (Supplementary Fig. 7f–i). These observations indicate that LoNA, but not mutant LoNA, modulates synaptic plasticity. Synaptophysin was used to indicate successful synaptosome isolation and prohibitin, an identifier for mitochondria, was included as a negative control (Supplementary Fig. 7j). We next examined dendritic spine structure from the pyramidal neurons of hippocampal CA1 region by Golgi staining and found that dendrites spine density was significantly increased in LoNA knockdown mice, decreased in LoNA-overexpressed mice, and largely unchanged in mutant LoNA-administered mice (Fig. 5h). Quantification analysis also supported this conclusion (Fig. 5h). We then measured long-term potentiation in the hippocampal CA1 region in vivo when the theta burst stimulus was evoked at the CA3 region. Consistent with Golgi staining, LoNA-administered mice displayed severe LTP deficits compared with control mice (Fig. 5i). Together, these results indicate a critical role for LoNA in maintaining neuronal integrity and function in adult brains. Direct injection of AAV containing LoNA shRNA into the CA1 of the hippocampus brain resulted in a significant increase of ribosomal protein (Fig. 5j) and ribosome RNAs including 45S, 28S, 18S, and 5.8S rRNA in synaptosome fractions (Fig. 5k). In addition, the enhanced ribosomal protein level was also observed in total hippocampal brain lysates (Supplementary Fig. 7k). However, the ratio of synaptosome fraction to total lysate still remained upregulated in LoNA hippocampal deficient mice (Supplementary Fig. 7l), indicating that local translational machinery is increased to accommodate the demand of local protein synthesis. On the other hand, the levels of ribosomal proteins, including Rps6, Rps3, Rpl23, and rRNAs, were significantly decreased in synaptosome fractions from LoNA-administered hippocampal brain (Supplementary Fig. 7m–n). However, neither ribosomal protein nor rRNA levels were influenced by mutant LoNA (Supplementary Fig. 7m–n). Ribosomes were further purified from synaptosomes of LoNA-deficient or LoNA-overexpressed brain, and ribosome-bound mRNAs were subjected to qPCR. These analyses indicated that ribosome-bound PSD95 and CamKII mRNA levels were significantly increased in the absence of LoNA, decreased in the presence of LoNA, and remained unaltered in the presence of mutant LoNA (Fig. 5l, m). Our findings demonstrate that some synapse plasticity-related proteins are locally translated, and that dendritic ribosome levels were increased to meet the translational demands.

### LoNA deficiency leads to improved learning and memory

**Fig. 6:**
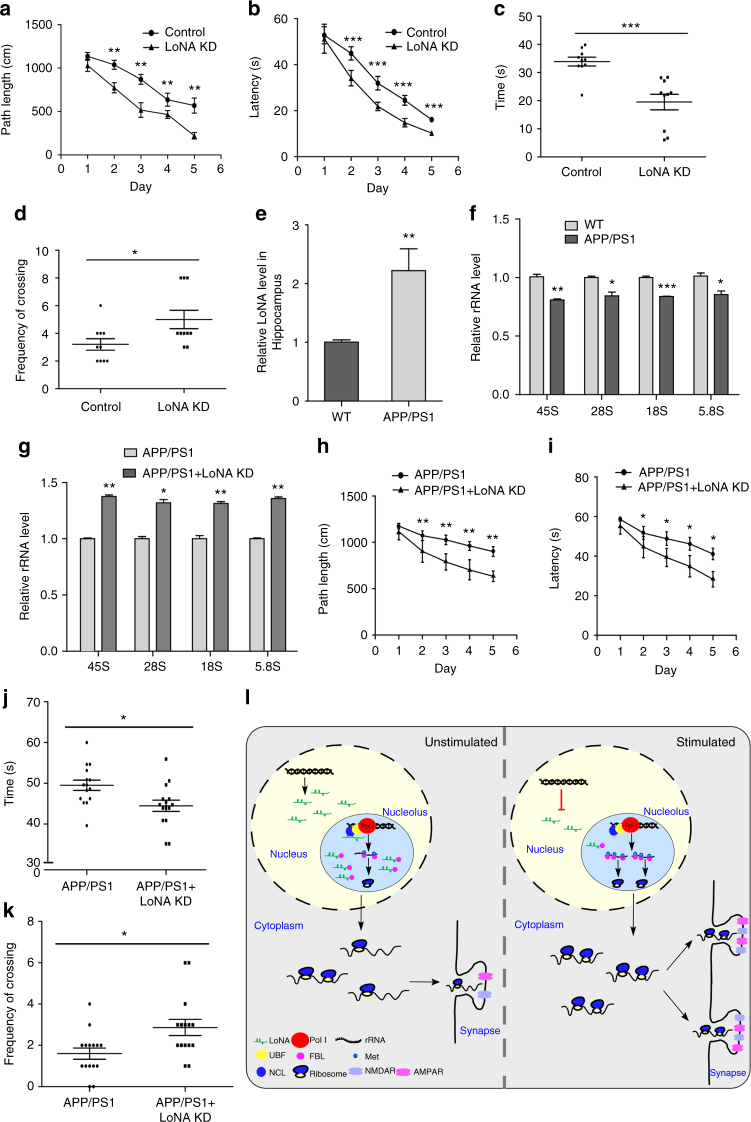
LoNA deficiency in hippocampal brain leads to enhanced learning and memory in WT mice, and restored learning and memory deficits in *APP/PS1* mice. **a**–**d** In the Morris water maze test, LoNA hippocampal knockdown mice entered the target quadrant with significantly lower travel distance (**a**) and less travel time (**b**) during the training phase (*n* = 10 for each group). These mice took significantly less time to locate the hidden platform (**c**) and more frequently crossed the target quadrant (**d**) during the probe trial (*n* = 10 for each group). **e** Expression levels of LoNA in the hippocampal brain of *APP/PS1* transgenic mice were increased, as determined by qPCR (age of 8 month, *n* = 7 for each group). **f** pre- and mature rRNAs in the hippocampal brain of *APP/PS1* transgenic mice were downregulated, as determined by qPCR (age of 8 months, *n* = 7 for each group). **g** Pre- and mature rRNA levels in the hippocampal brain of LoNA-deficient *APP/PS1* transgenic mice were restored, as measured by qPCR (age of 8 months, *n* = 10 for each group). **h**–**k** Hippocampal LoNA shRNA-administered *APP/PS1* mice exhibited improved behavior in Morris water maze test. These mice entered the target quadrant with significantly lower travel distance (**h**) and less travel time (**i**) during the training phase (age of 8 months, *n* = 10 for each group). These mice took significantly less time to locate the hidden platform (**j**) and more frequently crossed the target quadrant (**k**) during the probe trial (age of 8 months, *n* = 10 for each group). **l** Schematic representation for the proposed mechanism underlying LoNA-controlled translational regulation and rRNA biogenesis. In this figure, error bars, s.e.m.; *P < 0.05; **P < 0.01; ***P < 0.001 by ANOVA or two-tailed Student’s *t* test

We next conducted behavioral tests to evaluate learning and memory performance. Application of the Morris water maze test demonstrated that mice lacking LoNA took significantly less time and path to locate the hidden platform when compared to controls during the training phase (Fig. 6a, b). During the probe trial, LoNA knockdown mice entered the target quadrant, where the platform had been located during training, significantly more often and spent considerably less time locating this target quadrant compared to controls (Fig. 6c, d). Conversely, mice administered LoNA in the hippocampal brain exhibited impaired spatial learning and memory, and mice receiving the mutant LoNA behaved similarly to controls (Supplementary Fig. 8a–e). Decreased rRNA levels were observed in hippocampal brain of LoNA-recipient mice, but not mutant LoNA-recipient mice (Supplementary Fig. 8f). Application of the object-context discrimination test revealed that LoNA-deficient mice spent more time on object exploration in a novel context than control mice, whereas LoNA-overexpressed mice showed no preference and mutant LoNA mice exhibited no significant difference from controls (Supplementary Fig. 8g, h). These results suggest that hippocampal LoNA is deeply involved in learning and memory. Alzheimer’s disease (AD) patients exhibit decreased rRNA production^29^. Eight-month-old *APP/PS1* transgenic mice exhibit severe learning and memory impairment, and represent an animal model of AD. Interestingly, we observed an increased abundance of LoNA and reduced rRNAs in *APP/PS1* mice brain (Fig. 6e, f). These findings prompted us to investigate the function of LoNA in neurodegenerative diseases. We knocked down LoNA in hippocampal brain of *APP/PS1* transgenic mice by AAV delivery system, and performed Morris water maze and object-context discrimination behavioral tests. These studies indicated that LoNA-deficient *APP/PS1* mice showed rescued learning and memory deficits, when compared to control *APP/PS1* mice (Fig. 6h–k and Supplementary Fig. 8i). In addition, rRNA levels were found restored (Fig. 6g). Our results suggest that LoNA plays a key role in neurodegenerative diseases and may represent a promising therapeutic target for the treatment of AD.

Collectively, our results demonstrate that LoNA plays a key role in regulating rRNA transcription and post-transcriptional modification (Fig. 6l). Our data also reveal that LoNA serves as a sensor of neuronal activities and its levels are reduced rapidly upon neuronal stimulation, therefore leveraging rRNA production and subsequent protein synthesis, including synaptic proteins. Our data also uncover that suppression of LoNA improves learning and memory in WT mice, and rescues impaired learning and memory in *APP/PS1* mice. These observations improve our understanding of and provide opportunity for the treatment of neurodegenerative diseases.

## Discussion

Ribosome biosynthesis, which takes place in nucleoli, is a major cellular undertaking. Multiple mechanisms are employed to modulate the rate of ribosomal production to meet cellular demands^30^. The primary target of regulation is rDNA transcription. rRNA synthesis accounts for the majority of transcriptional activity to accommodate ribosome production and protein synthesis, it is required for the maintenance of long-term synaptic plasticity in central nervous system^31^. Nucleolar remodeling complex (NoRC) associated RNA (pRNA) silences rRNA transcription by interacting with TIP5. However, a more comprehensive characterization of long non-coding RNAs and their regulatory roles in nucleoli remains elusive. By high-throughput sequencing, our work identifies a nucleolar lncRNA LoNA, which is highly expressed in neurons, also evident by single-cell RNA sequencing^32^. Levels of LoNA are relatively low in astrocytes, and critically low in microglial cells^33^. This suggests a critical role of LoNA in neuronal functions. Our work shows that LoNA is specifically localized to nucleoli and regulates rRNAs production and ribosome assembly. The copy number (CN) of LoNA is 4–6 times greater than the CN of rDNA in variety of cells, suggesting that the amount of LoNA is sufficient to inhibit rDNA transcription. LoNA is not conserved across species in terms of sequences. However, we did identify a human lncRNA RP11-517C16.2, which only shares identical sequences with LoNA on NCL and FBL-binding sites, indicating that RP11-517C16.2 maybe functionally similar to LoNA. According to GTEx Portal (v7), RP11-517C16.2 is ubiquitously expressed in human tissues, with relatively high levels in brain tissue. We further examined its expression level in different human cell lines, and found that it is abundantly expressed in SH-SY5Y cells, and barely expressed in 293T, U2OS, and U87 cells (Supplementary Fig. 8j).

NCL is the major nucleolar protein of growing eukaryotic cells. It possesses an acidic domain on the N terminus, RNA recognition motifs (RRMs) on the central domain, and a glycine/arginine-rich domain (GAR/RGG) on the C terminus. Acidic sequences interact with histone H1 and induce chromatin decondensation^19,34^. NCL’s central domain possesses four RNA-binding motifs and is implicated in various functions^35^. The expression of NCL is highly correlated with rRNA level and proliferative activity of the cell^36,37^. The depletion of NCL induces inhibition of Pol I transcription in mammalian and chicken cells^38,39^, thereby hindering the transcription of rDNA^30,40^. Histone modifications distinguish silent heterochromatin from permissive euchromatin and tightly correlate with the transcription of rDNA. Reduced expression of NCL in HeLa cells results in increased H3K9m2 and decreased H3K4m3, ultimately leading to rDNA silencing[41]. Our work demonstrates that LoNA, harbors NCL binding sites on its 5′ portion, directly binds to NCL, and sequesters NCL activity. This consequently reduces UBF and polI loading on rDNA chromatin and alters the epigenetic state of rDNA, ultimately inhibiting rRNA transcription.

rRNAs generally carry chemical modifications, including base methylation, pseudo-uridylation, and ribose methylation at the 2′-hydroxyl (2′-*O*-methylation). The most abundant modification, 2′-*O*-methylation, is catalyzed by FBL. Interestingly, rRNAs' methylation generally cluster around functional ribosomal sites, including the decoding site and peptidyl transferase center, and are believed to modulate ribosome functions. Genetic studies also demonstrated that 2′-*O*-methylation is indeed essential for the translation capacity of ribosomes[42]. We demonstrate that LoNA binds to FBL competitively with U3 through box C/D sequence on its 3′ portion, resulting in diminished FBL activity and decreased 2′-*O*-methylation of rRNAs. In our study, we further evaluated rRNA methylation at sites localized within key functional domains of rRNAs: the decoding center (DC) in the 18S rRNA, the peptidyl transferase center (PTC), and the helix 69 (H69) of 28S rRNA. We found that most of these sites were significantly less frequently methylated in the presence of LoNA, which is consistent with attenuated activity of FBL. rRNA heterogeneity mainly originates from differential rRNA processing and modification. Our study shows only subtle changes in rRNA processing were observed, implying that rRNA methylation, rather than processing, is the primary driver of heterogeneity. Our work is, to our knowledge, the first to demonstrate that a nucleolar-specific lncRNA, LoNA, uniquely serves as a dual modulator of both rRNAs transcription and methylation, elucidating the role of ncRNAs in the regulation of rRNA biosynthesis.

Synaptic plasticity is one of the most important neurochemical foundations of learning and memory, serving to strengthen or weaken synapses in order to increase or decrease their activities. In the adult brain, eliciting appropriate responses to stimuli requires precisely regulated protein synthesis. Thus, dedicated regulatory mechanisms are adopted to meet these requirement in the process of functional protein production. Many of these mechanisms target mRNA-binding proteins and ribosomal subunits to regulate translational initiation. However, regulation of ribosome number and/or function is essential to meet the demands of protein synthesis as well. In this study, we find that the lncRNA LoNA is abundantly expressed to nucleoli and inhibits rRNA production by inhibiting the activity of NCL and FBL. LoNA levels are highly sensitive to neuronal activity, as its expression drops rapidly in response to stimulation, alleviating the inhibition of rRNA production. This eventually leads to elevated protein synthesis including AMPA/NMDA receptors, enhanced synaptic plasticity, LTP, and improved learning and memory (as demonstrated in both WT and *APP/PS1* mice).

Deep RNA sequencing revealed that a great number of mRNAs are dendritically localized^10^. Evidences suggest that some are translated locally at synapses, including calcium/calmodulin-dependent kinase IIα (CaMKIIα)^43,44–46^, Arc/Arg3.1^47^, glutamate receptor 1 (GluR1), and GluR2^48,49^. Synaptic activity induces polyribosomes to migrate to the base and necks of dendritic spines^11^, implying that translation machinery is enhanced to meet the demands of protein synthesis at synapses. Our work demonstrates that LoNA deficiency leads to a boost of both rRNA and ribosomal proteins at synapse, elevated levels of PSD95, CamKIIa, AMPA receptors, and NMDA receptors locally, ultimately leading to improved neuronal plasticity and LTP.

Impaired nucleolar activity, also known as nucleolar stress, defined as the impairment of rDNA transcription and disruption of nucleolar integrity, are primary signs of cellular stress associated with aging and neurodegenerative diseases. Silencing of rDNA occurs during the early stages of AD pathology, which appears to account for AD-related ribosomal deficiency^50^. Moreover, AD patients exhibited a reduction of ribosomal gene activity and decreased level of mature rRNA 28S/18S ratio^51,52^. Neuronal nucleoli show significantly hypertrophic changes in cortical brain of asymptomatic AD patients^53^, implying that nucleoli impairment could be an early characteristic of these diseases. This report demonstrates that LoNA is upregulated in *APP/PS1* transgenic mice, and a reduction of LoNA levels in the hippocampus of these mice rescues their learning and memory deficits, illustrating that LoNA is implicated in neurological diseases and may provide a novel avenue for treatment of neurodegenerative disorders.

## Methods

### Materials

N2a cell originated from the ATCC and was cultured under standard conditions with DMEM plus 10% FBS and 1% penicillin/streptomycin (Invitrogen) at 37 °C under 5% CO_2_. Cell lines tested negative for mycoplasma contamination. In all of the in vivo experiments, mice were randomly allocated into experimental groups, and the experimenter was blinded in regard to the applied treatments. Variation within groups allowed the detection of differences with 6–10 mice per group. Protocols involving C57BL/6J mice were approved by the Institutional Animal Care and Use Committee at the University of Science and Technology of China.

The list of used antibodies is reported in Supplementary Information (Supplementary Table 1). TUNEL FITC Apoptosis Detection Kit (A111) was purchased from Vazyme, RNA Polymerase I inhibitor (CX5461) from TargetMol, RNA Polymerase II inhibitor α-Amanitin (HY-19610) from MCE, RNA Polymerase III inhibitor (sc-222257) from Santa Cruz Biotechnology. Peroxidase-labeled anti-mouse or anti-rabbit antibody were purchased from GE Healthcare and ECL System was from Thermo Scientific. Fluorescence-labeled anti-mouse or anti-rabbit antibodies were from Invitrogen. Uncut blots are supplied in Supplementary Figs. 9–15.

### Fluorescence in situ hybridization (FISH) and immunostaining

RNA probes for LoNA were in vitro transcribed using T7 High Yield Transcription Kit (Thermo Scientific), and then labeled with Alexa Fluor 488 or Alexa Fluor 594 on every G according to the manufacturer’s protocol (ULYSIS Nucleic Acid Labeling Kit, Invitrogen). RNA probes and cells were denatured at 80 °C for 10 min, and then incubated with probes for 24 h at 42 °C, followed by 2× SSC washing for 10 min at 45 °C. For immunostaining, slides after FISH were incubated with anti-NCL, anti-FBL, or anti-PolR1E antibodies for 3 h at 37 °C. Signals were detected and visualized using Alexa 594 or Alexa 488-labeled secondary antibodies (Invitrogen).

### Northern blots and densitometry analysis

RNA probes were generated using T7 RNA polymerases by in vitro transcription, DNA probes were synthesized at Sangon Biotech (Shanghai). The 3′ end of probes was biotinylated using terminal deoxynucleotidyl transferase (TDT) from Invitrogen. Total RNAs and RiboRuler High Range RNA Ladder (Thermo Scientific) were resolved on agarose gels containing 1% formaldehyde, and then capillary transferred and UV crosslinked onto a positively charged NC membrane (Millipore). Hybridization of RNA and biotinylated probes was performed at 42 °C overnight; signals were further amplified by HRP-streptavidin and visualized by enhanced chemiluminescence (ChemiScope, CLiNX). Immunoreactive bands were quantified using ImageJ software for densitometric analyses. Probes for 45S, 41S, 34S, 36S, 28S, 18S, 5.8S, and 5S are listed in Supplementary Table 2.

### RNA pulldown

Sample preparation for RNA pulldown was carried out as described previously^54^, with modifications. LoNA DNA probes, including eight antisense and eight sense probes, were synthesized by Sangon Biotech (sequences are listed in Supplementary Table 2). The 3′ end of each probe was biotinylated using TDT from Invitrogen. Cells were harvested and cross-linked in PBS containing 1% glutaraldehyde at RT for 10 min on an end-to-end shaker, then quenched in 0.125 M glycine at RT for 5 min. Cells were pelleted by centrifugation at 2000 × *g* for 5 min and lysed in buffer containing 50 mM Tris pH 7.0, 10 mM EDTA, 1% SDS, complete proteinase inhibitor (Roche), and RNase inhibitor (Vazyme) for 20 min on ice, then subjected to sonication for 10 min or until the lysate turns clear. The supernatant was saved after centrifugation and pre-cleared with M-280 Streptavidin Dynabeads (Life Technologies). A small amount of pre-cleared samples were saved as input as the remaining was incubated with biotinylated antisense or sense oligos at 4 °C overnight. M-280 streptavidin magnetic Dynabeads (Invitrogen) were added to the mixture and incubated for another 30 min at 37 °C with rotation. Beads were captured by magnets (Life Technologies) and washed five times with buffer (300 mM Nacl, 30 mM sodium citrate, 0.5% SDS, PMSF, Roche cocktail proteinase inhibitor, SUPERaseIn RNase inhibitor). RNAs and proteins were eluted (12.5 mM biotin [Invitrogen], 7.5 mM HEPES pH 7.5, 75 mM NaCl, 1.5 mM EDTA, 0.15% SDS, 0.075% sarkosyl, and 0.02% Na-Deoxycholate) from beads for further analysis.

### Nuclear run-on (NRO) analysis

NRO was carried out according to a published protocol described by Roberts et al.^55^, with modifications. For nuclei isolation, cells were washed with PBS three times, trypsinized and collected in NP-40 lysis buffer (10 mM Tris-HCl pH 7.4, 10 mM NaCl, 3 mM MgCl2, 0.5% NP-40). Pelleted were harvested by centrifugation and resuspended in nuclei storage buffer (50 mM Tris-HCl pH 8.3, 0.1 mM EDTA, 5 mM MgCl_2_, 40% (vol/vol) glycerol). The nuclear run-on mixture (10 mM Tris-HCl pH 8.3, 2.5 mM MgCl_2_, 150 mM KCl, 2 mM DTT, 100 U RNaseOUT (40 U µl^−1^), 1 mM ATP, 1 mM CTP, 1 mM UTP, 1 mM GTP, 200 µM 4-Thiouridine, and the crude nuclei) was incubated at 30 °C for 15 min. Total RNA was isolated with TRIzol (Invitrogen) and resuspened in ddH_2_O. NRO-RNA was biotinylated by EZ-link HPDP-biotin (Pierce) at RT for 1.5 h and captured with Dynabeads Streptavidin beads (Invitrogen). Beads bounded NRO-RNA was eluted and subjected to qPCR analyses.

### Data availability

Data that support the findings of this study have been deposited in the NCBI Gene Expression Omnibus (GEO; http://www.ncbi.nlm.nih.gov/geo/) with the accession number GSE110016 and all relevant data are available from the authors upon reasonable request.

## Electronic supplementary material

Supplementary Information
