# Supplementary material for: Activity dependent LoNA regulates translation by coordinating rRNA transcription and methylation
Source: Nat Commun. 2018 Apr 30;9:1726. doi: 10.1038/s41467-018-04072-4 (PMC5928123; doi:10.1038/s41467-018-04072-4)
Supplement: Supplementary file 1 — Supplementary Information [file 41467_2018_4072_MOESM1_ESM.pdf]

SUPPLEMENTARY INFORMATION

Activity dependent LoNA Regulates Translation by Coordinating rRNA Transcription and Methylation

Li et al.

Supplementary Figure 1

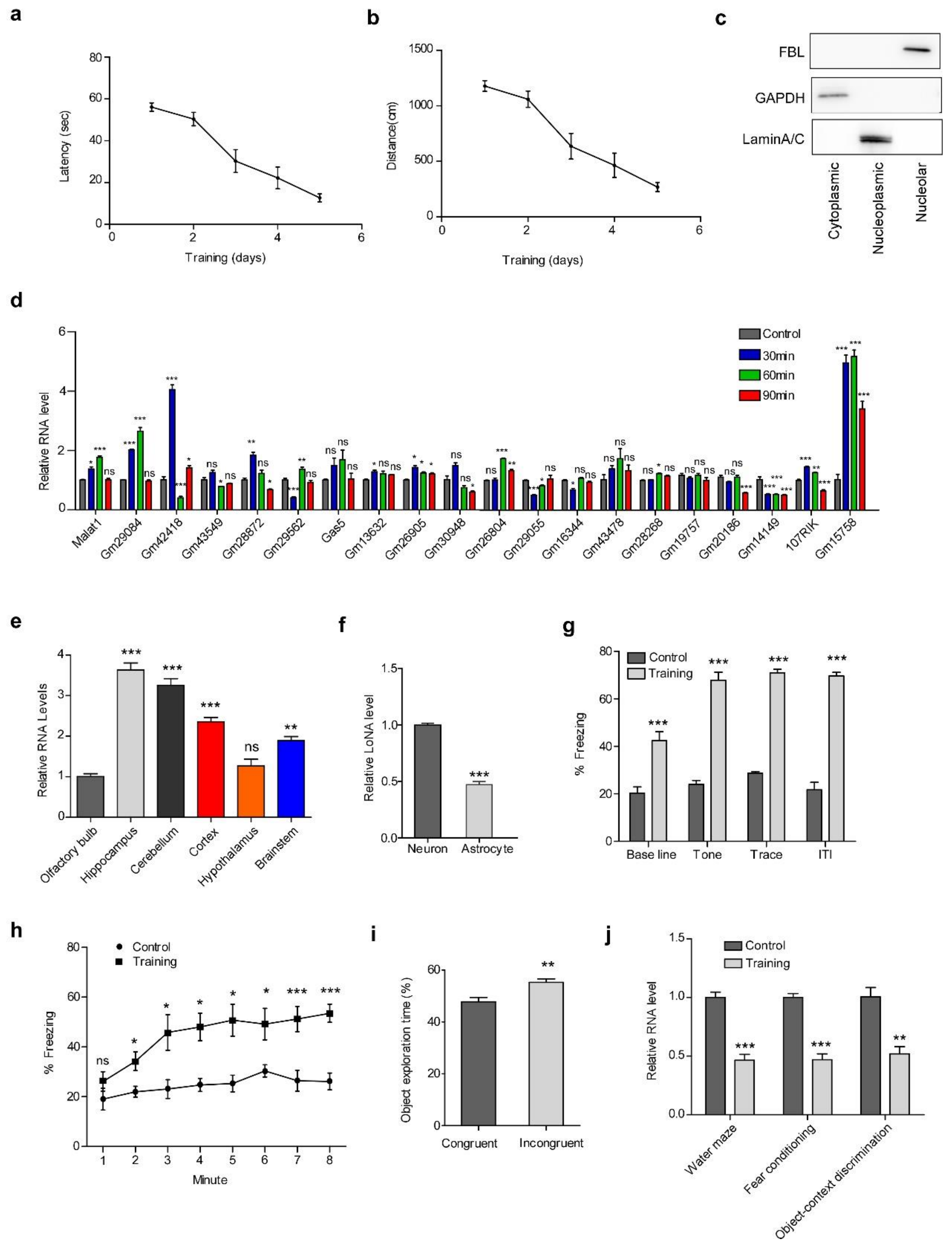

Supplementary Figure 1| (**a-b**), During the training phase of Morris water maze task, spatial learning was assessed in WT C57BL/6J mice as a function of training day with respect to the following parameters: (**a**) escape latency, (**b**) total path length to the target quadrant. Data were presented as the means ± s.m.e. (n=10). WT mice entered the target quadrant with significantly lower travel time and less travel distance, indicating a successful spatial learning. (**c**), Cellular fractionation. Representative

Western blots using antibodies raised against the cell fraction-specific markers FBL (nucleolus), GAPDH (cytoplasm) and lamin A/C (nucleus) to indicate the purity of the nucleolar extracts. (**d**), lncRNA (validated in Fig1d, but not shown in Fig1f) levels in KCL activated primary neurons at different time point. (**e**), LoNA expression pattern across different brain regions. (**f**), LoNA levels were determined in different cell types of mouse brain, including primary cultured neurons and astrocytes, by qPCR. (**g-h**), Fear conditioning test. (**g**), the percent freezing for C57BL/6J mice during the tone test following trace fear conditioning. Baseline, trace, and intertribal interval (ITI) were included. (**h**), the percent freezing for mice during the context test. A 8 min trial of context condition testing was conducted 48 h after trace conditioning was implemented. (**i**), The object-context discrimination test. A 10 min trial of object and context condition testing was conducted. (**j**), LoNA expression levels were examined in hippocampal brain of mice subjected to the Morris water maze, Fear conditioning or Object-context discrimination training respectively, as determined by qPCR and normalized against U1 snRNA. Error bars, s.e.m.; *P < 0.05; **P < 0.01; ***P < 0.001 by ANOVA or two-tailed Student's t test.

Supplementary Figure 2

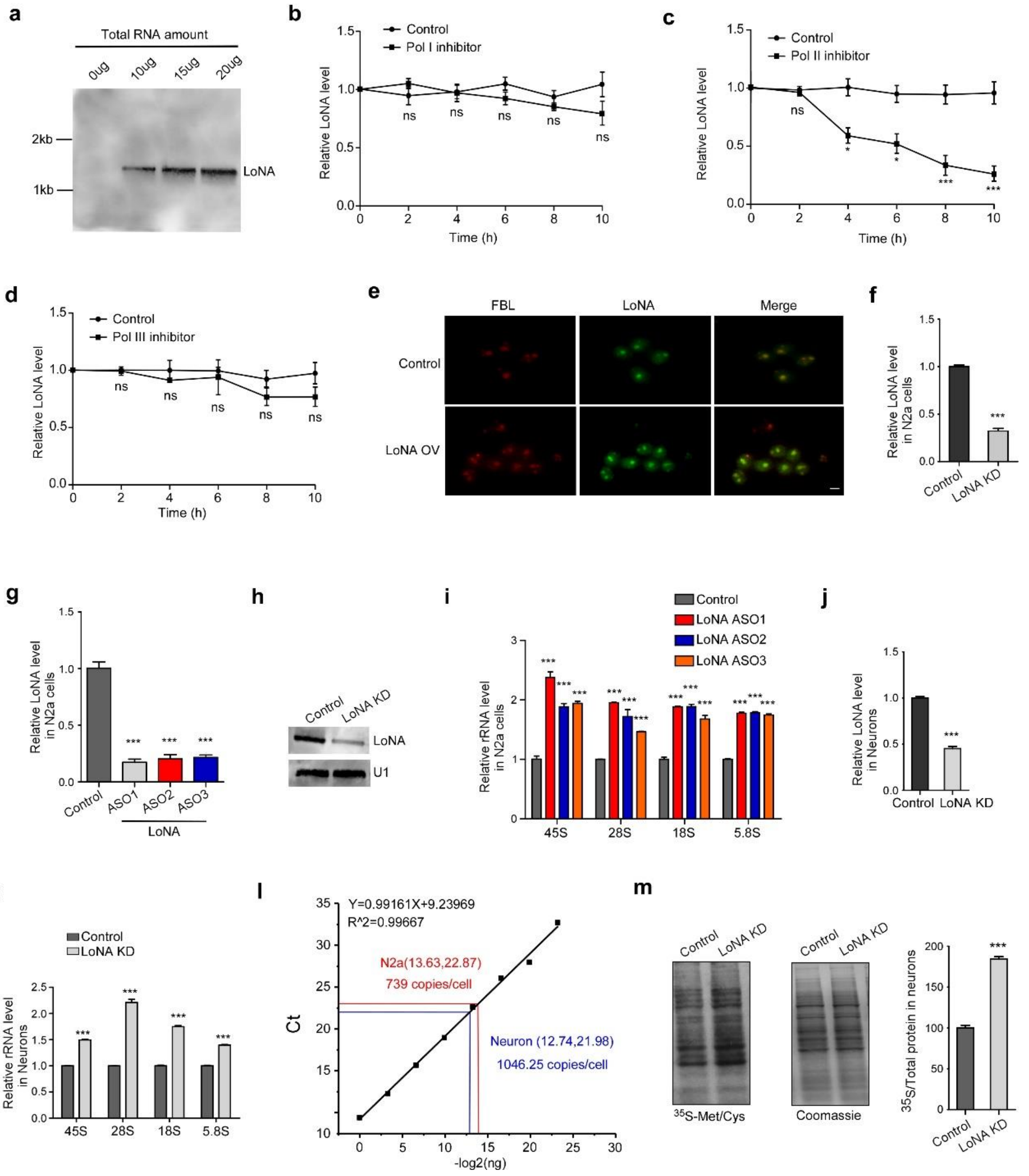

Supplementary Figure 2| (**a**), Northern blot detection of LoNA in N2a cells with regard to different amount of total RNA. (**b**), N2a cells were subjected to the treatment of 142 nM polI inhibitor or (**c**) 10 μg ml^{-1} polII inhibitor α-Amanitin or (**d**) 10 μg ml^{-1} polIII inhibitor, total RNA was collected at various time point, and LoNA levels were determined by qPCR. Data were normalized to the level at time point 0. (**e**) Representative immunofluorescence images of FBL (red) and LoNA RNA FISH (green) in control and N2a cells with LoNA overexpression. Scale bar: 10 μm. (**f**), LoNA expression was assessed in N2a cells with shRNA knockdown, as determined by qPCR analyses. Data were normalized to U1 snRNA level. (**g**), LoNA expressions

were determined in N2a cells treated with three independent antisense oligos (ASO), as measured by qPCR analysis. Data are normalized to U1 snRNA level. (**h**), LoNA expression was assessed in N2a cells with shRNA knockdown, as determined by Northern blot, U1 snRNA was included as an input control. (**i**), Levels of pre-rRNA (45S) and mature rRNAs (28S, 18S, 5.8S) in N2a cells treated with three independent LoNA antisense oligo or control sequence were determined by qPCR and normalized against U1 snRNA level. (**j**), LoNA expression was determined in primary neurons with LoNA knockdown by shRNA, as measured by qPCR. (**k**), rRNA (45S, 28S, 18S, 5.8S) levels were enhanced in LoNA knockdown neurons, as determined by qPCR. Data were normalized against U1 snRNA level. (**l**), Plot of LoNA copy numbers in N2a cells and primary cultured neurons. (**m**), LoNA knock down and control primary neurons were pulsed with ^{35}S-Met/Cys, cell lysates were resolved on SDS-PAGE. *De novo* protein synthesis was quantified as the mean ratio of ^{35}S incorporation relative to total protein (Coomassie). Protein synthesis rate in LoNA knockdown neurons was normalized against control neurons. Error bars, s.e.m.; *P < 0.05; **P < 0.01; ***P < 0.001 by ANOVA or two-tailed Student's t test.

Supplementary Figure 3

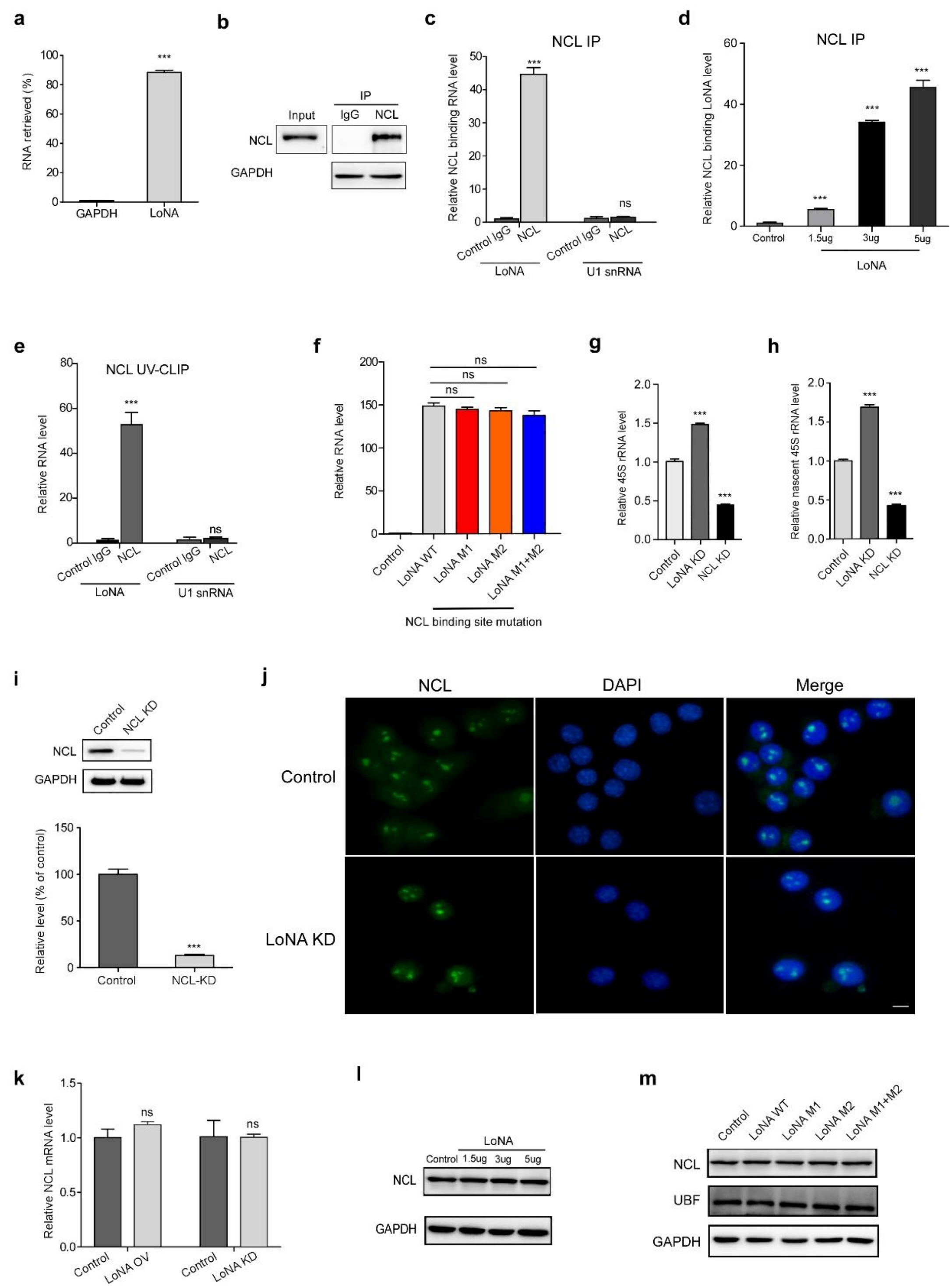

Supplementary Figure 3| (**a**), N2a cells were pulled down with biotinylated LoNA antisense DNA probes or control probes with sense DNA sequence, and LoNA level was determined by qPCR. (**b**), N2a cell lysates were subjected to immunoprecipitation with NCL antibody or control IgG, detected with NCL antibody. GAPDH was measured in total lysates to indicate equal input. (**c**), LoNA, but not U1,

was specifically pulled down by anti-NCL antibody. NCL bound RNA level was determined by qPCR. (**d**), N2a cells were introduced with various amount of LoNA, cell lysates were subjected to immunoprecipitation with NCL antibody, total RNA was then subjected to qPCR analyses. (**e**), UV-CLIP assay was conducted with anti NCL antibody, NCL bound LoNA fragment was detected by qPCR with specific primer pair. U1 was included as a negative control. Data were normalized to control IgG. (**f**), N2a cells were transfected with WT or mutant LoNA respectively, total LoNA levels were determined by qPCR. (**g**), Pre-rRNA 45S level was determined by qPCR in N2a cells transfected with NCL or LoNA shRNA, 45S level was normalized to U1. (**h**), Nascent 45S rRNA levels weres measured by nuclear run-on analysis in N2a cells transfected with NCL or LoNA shRNA, 45S levels were normalized to U1. (**i**), NCL knockdown by shRNAs in N2a cells, shown by Western blot and densitometric analyses. (**j**), Representative immunofluorescence images of NCL (green) and DAPI (blue), in control or N2a cells with LoNA knockdown. Scale bar: 10 µm. (**k**), NCL mRNA levels were examined in N2a cells with LoNA overexpression or knockdown, by qPCR. Data were normalized to GAPDH and plotted against to their respective controls. (**l**), NCL levels were not altered by an increased amount of LoNA. N2a cells were introduced with various amount of LoNA, cell lysates were then subjected to immunoblotting with NCL antibody. (**m**), NCL levels were not altered by WT nor mutant LoNA. N2a cells were introduced with same amount of WT or mutant LoNA, cell lysates were then subjected to immunoblotting with NCL or UBF antibody. Error bars, s.e.m.; *P < 0.05; **P < 0.01; ***P < 0.001 by ANOVA or two-tailed Student's t test.

Supplementary Figure 4

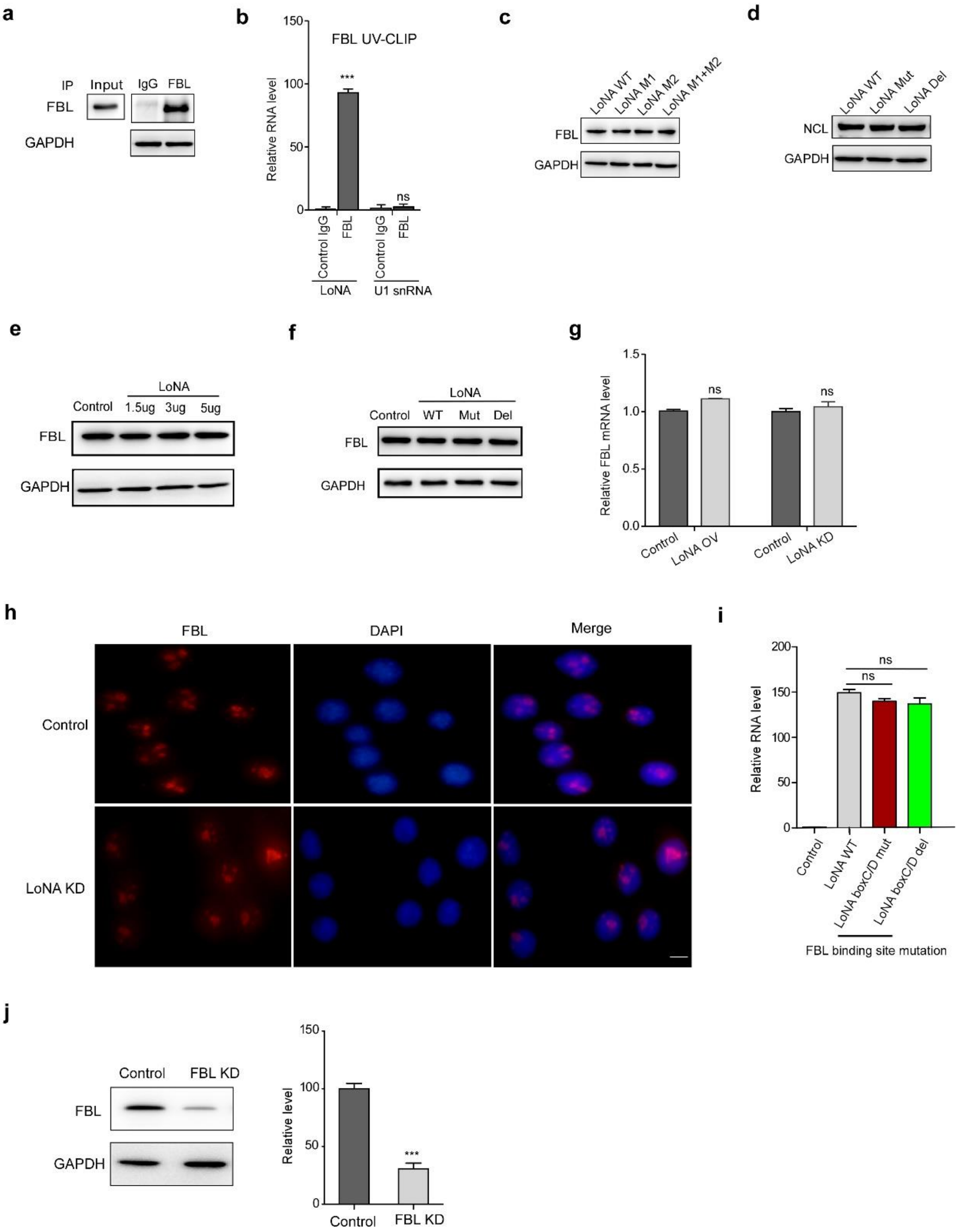

Supplementary Figure 4| (**a**), N2a cell lysates were subjected to immunoprecipitation with FBL antibody or control IgG, followed by SDS-PAGE and immunoblotting detection with FBL antibody. A small amount of lysates were used as input, and GAPDH was included to indicate equal input. (**b**), UV-CLIP assay was conducted with anti FBL antibody, FBL bound LoNA fragment was detected by qPCR with specific primer pair. Data were normalized to control IgG. U1 snRNA was included as a negative control. (**c**), Binding affinities of FBL to M1 or M2 or M1+M2

LoNA were determined by pulldown of biotinylated LoNA. GAPDH was included as an input control. (**d**), Binding affinities of NCL to Mut or Del LoNA were determined by pulldown of biotinylated LoNA. GAPDH was included as an input control. (**e**), FBL levels were not altered by increased amounts of LoNA. N2a cells were introduced with various amount of LoNA, a plasmid without target sequence was included as a negative control. Cell lysates were then subjected to immunoblotting with FBL antibody. GAPDH was included to indicate equal input. (**f**), FBL levels in N2a were not affected by introduction of WT, Mut, or Del LoNA, as measured by Western blot. (**g**), FBL mRNA levels were examined in N2a cells with LoNA overexpression or knockdown, by qPCR. Data were normalized to GAPDH and plotted against to their respective controls. (**h**), Representative immunofluorescence images of FBL (red) and DAPI (blue) in N2a cells with LoNA deficiency. Scale bar: 10 µm. (**i**), N2a cells were transfected with WT, box C/D del or box C/D mut LoNA respectively, total LoNA levels were determined by qPCR. (**j**), FBL knockdown by shRNA in N2a cells, shown by Western blot and densitometric analyses, GAPDH was used to indicate equal input. FBL levels in knockdown cells were normalized against control shRNA. Error bars, s.e.m.; *$P < 0.05$; **$P < 0.01$; ***$P < 0.001$ by ANOVA or two-tailed Student's t test.

Supplementary Figure 5

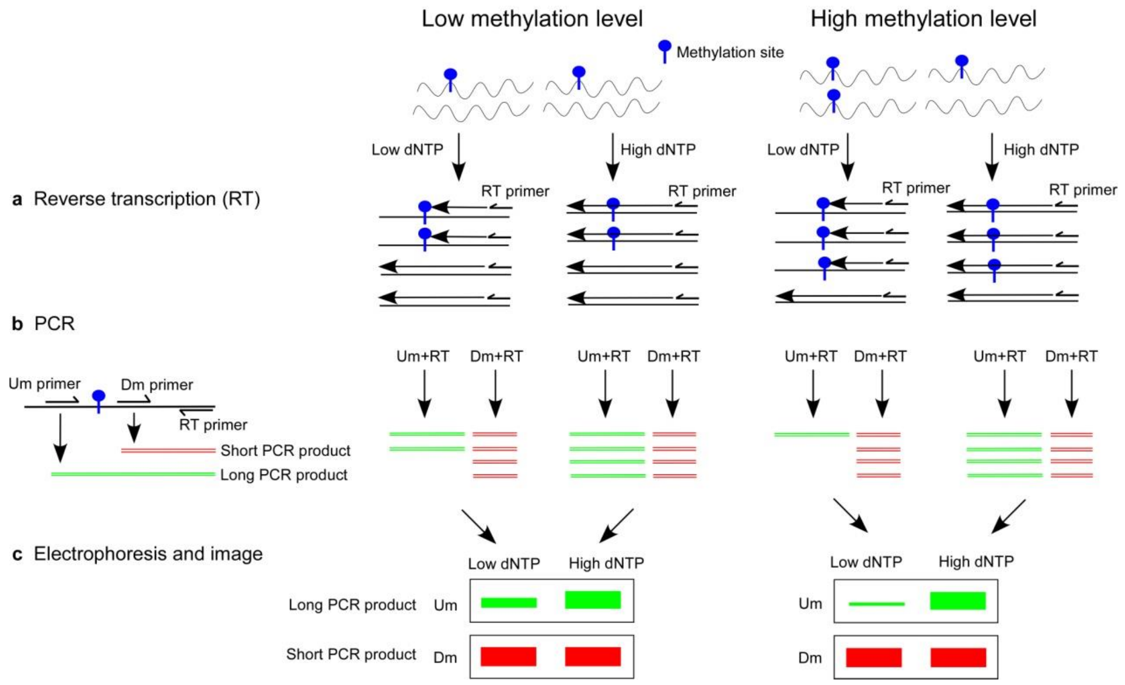

Supplementary Figure 5| A schematic of RTL-P approach for detection of the presence of 2'-O-methylation in RNA. (**a**), RT reaction was conducted with RT primer at low (1μM) or high (1mM) concentration of dNTP respectively. At high level of dNTP, the RT reaction produce long RT products (denoted in green color), at low level of dNTP, the RT reaction is impeded by the 2'-O-methyl groups, leading to a shorter RT products (denoted in red color). (**b**), PCR with different PCR primer pairs targeting upstream (Um) or downstream (Dm) of a specific methylation site. For RNAs with high level of methylation sites, the quantity of the RT primer/Um primer PCR products is less than those RNAs with low level of methylation sites in presence of low concentration of dNTP. This quantity is similar in presence of high concentration of dNTP. (**c**), Electrophoresis to indicate the size and intensity of PCR products.

Supplementary Figure 6

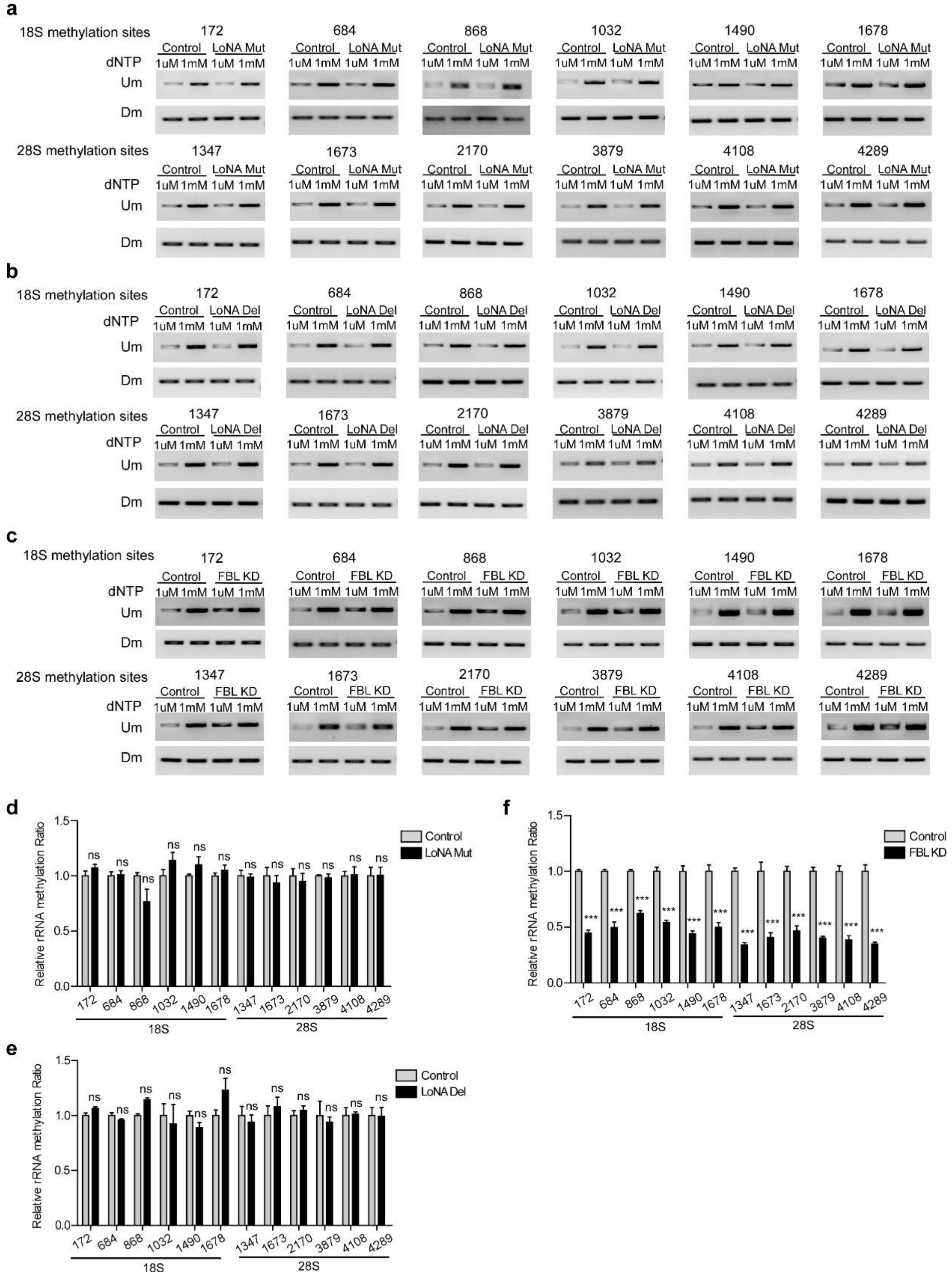

Supplementary Figure 6| (**a-c**), The detection of 12 methylation sites distributed throughout the 18S and 28S rRNA by RTL-P. Six sites were determined for the 18S, and other six for the 28S. Total RNAs from N2a cells containing LoNA box C/D mut (**a**), or LoNA box C/D del (**b**), or FBL shRNA (**c**) were subjected to RT with low (1μM) or high (1mM) concentration of dNTP respectively. cDNA was then amplified

with primer pair corresponding to upstream (Um) or downstream (Dm) of a specific methylation site. (**d**-**f**), In RTL-P assay, data were presented as signal intensity ratio of amplification products at low dNTP (1μM) over high dNTP (1mM) level. Methylation ratio in LoNA box C/D mut N2a cells were normalized to its control N2a cells (**d**), in LoNA box C/D del cells were normalized to its control N2a cells (**e**), and in FBL knockdown cells were normalized to cells containing control shRNA (**f**). Position of the nucleotide that is analyzed was indicated in x-axis. Error bars, s.e.m.; *P < 0.05; **P < 0.01; ***P < 0.001 by ANOVA or two-tailed Student's t test.

Supplementary Figure 7

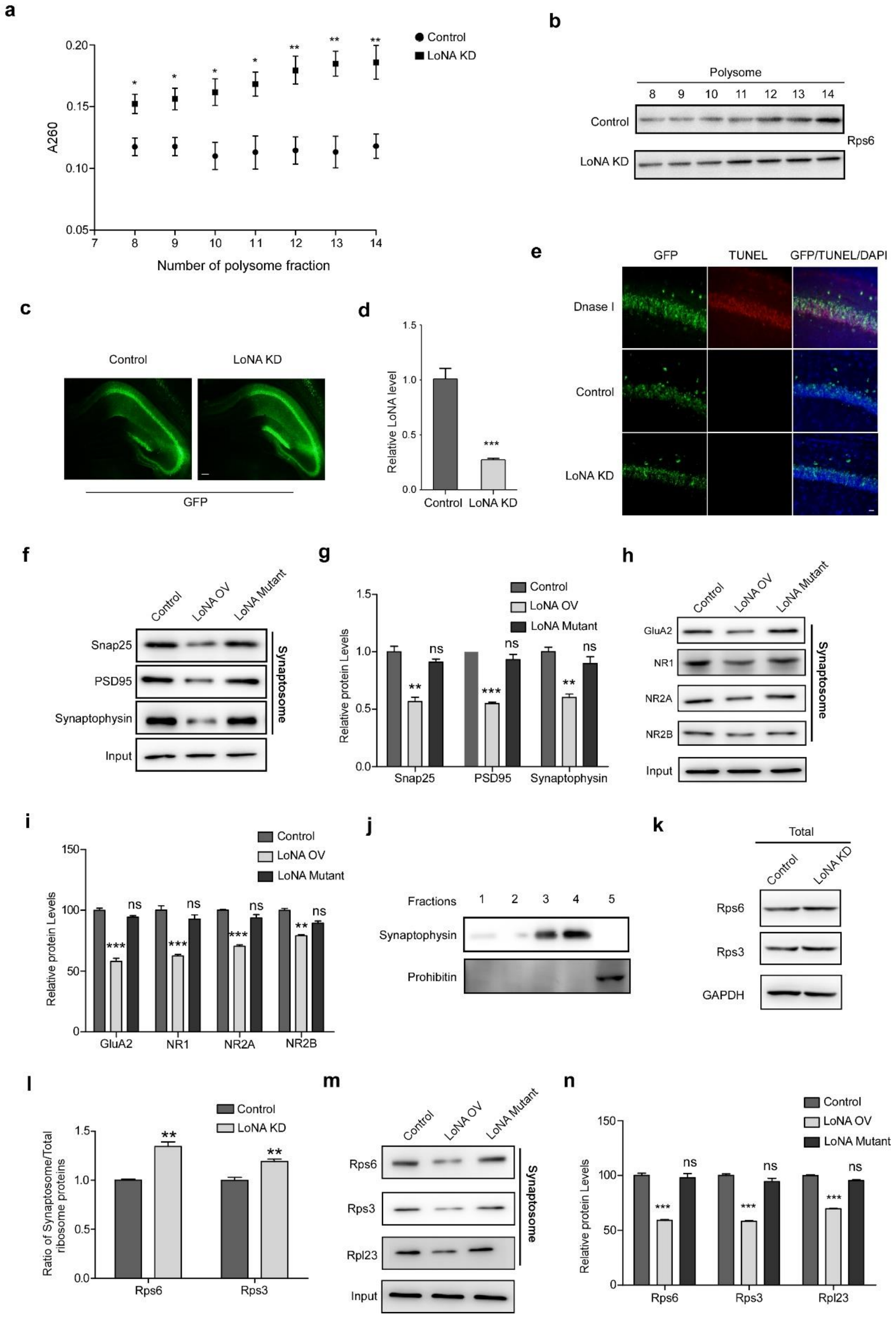

Supplementary Figure 7| (**a**), Readings for each polysomal fractions from 3 independent ribosome profiling were averaged and plotted. (**b**), Rps6 levels were

determined in each polysome fraction of LoNA deficient or control N2a cells to indicate successful fractionations, as measured by western blot. (**c-d**), LoNA was knocked down *in vivo* using an AAV vector (containing GFP element) in the hippocampus of C57BL/6J WT mice. (**c**), a representative image of LoNA knockdown in hippocampal brain. Scale bar: 100 µm. (**d**), LoNA level in injected hippocampal brain was determined by qPCR. (**e**), Representative images of TUNEL staining on AAV injected brain sections, GFP (green), apoptotic signal (red) and DAPI (blue). Dnase I treatment was included as a positive control. Scale bar: 20 µm. (**f-i**), Synaptosome fractions were purified from hippocampal brain of control, LoNA or mutant LoNA administered mice. (**f-g**), Levels of synaptic protein PSD95, snap25 and synaptophysin were determined by western blot and subsequent densitometric analysis. Equal amounts of brain tissues were used and GAPDH was measured as an input control. (**h-i**), Levels of AMPA receptor GluA2, NMDA receptor NR1, NR2A and NR2B were measured by western blot and densitometric analysis. Equal amounts of brain tissue were used and GAPDH was determined as an input control. (**j**), Levels of synaptophysin were determined by western blot in synaptosome isolated from WT hippocampal brain to indicate a successful isolation. Mitochondria marker prohibitin was included as a negative control. (**k**), Ribosomal protein levels were determined in total hippocampal brain lysates, by western blot. GAPDH level was determined as an input control. (**l**), Ratio of ribosomal proteins in synaptosomes fractions and total lysates, by densitometric analysis. (**m-n**), Levels of ribosomal protein Rps6, Rps3 and Rpl23 were determined by western blot (**m**) and densitometric analysis (**n**) in synaptosome fractions of LoNA or mutant LoNA administered mice. Equal amounts of brain tissue were used and GAPDH was measured as an input control. Error bars, s.e.m.; $^*P < 0.05$; $^{**}P < 0.01$; $^{***}P < 0.001$ by ANOVA or two-tailed Student's t test.

Supplementary Figure 8

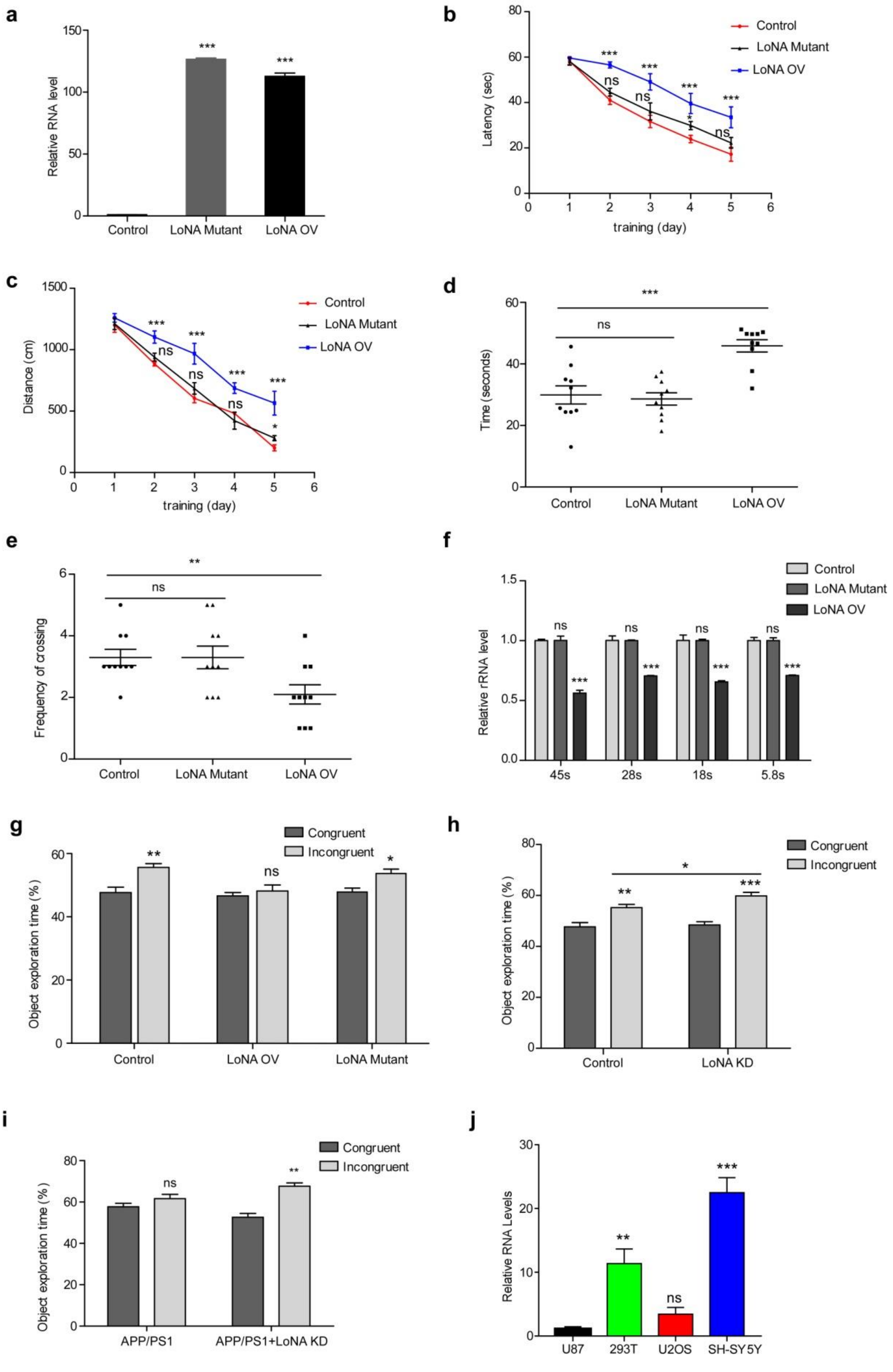

Supplementary Figure 8| (**a**), LoNA or mutant LoNA neuronal overexpression *in vivo* using an AAV vector in the hippocampus of C57BL/6J WT mice. (**b-e**), Morris water maze behavioral test of LoNA or mutant LoNA hippocampal overexpression and control mice. Target quadrant entering time (**b**) and travel distance (**c**) were assessed during the training phase (n=10 for each group). Time to locate the hidden platform (**d**) and frequency crossing the target quadrant (**e**) were determined during the probe trial (n=10 for each group). (**f**), pre- and mature rRNA levels of LoNA hippocampal injected and control mice were analyzed by qPCR, data were normalized against U1 snRNA. (**g-i**), Pattern separation memory was assessed in an object-context discrimination test of WT mice administered with LoNA or mutant LoNA (**g**), LoNA shRNA (**h**), of *APP/PS1* transgenic mice administered with LoNA shRNA (**i**). (**j**), Levels of RP11-517C16.2 in variety of human cells, as determined by qPCR. Data were normalized to GAPDH. Error bars, s.e.m.; *P < 0.05; **P < 0.01; ***P < 0.001 by ANOVA or two-tailed Student's t test.

Supplementary Figure 9

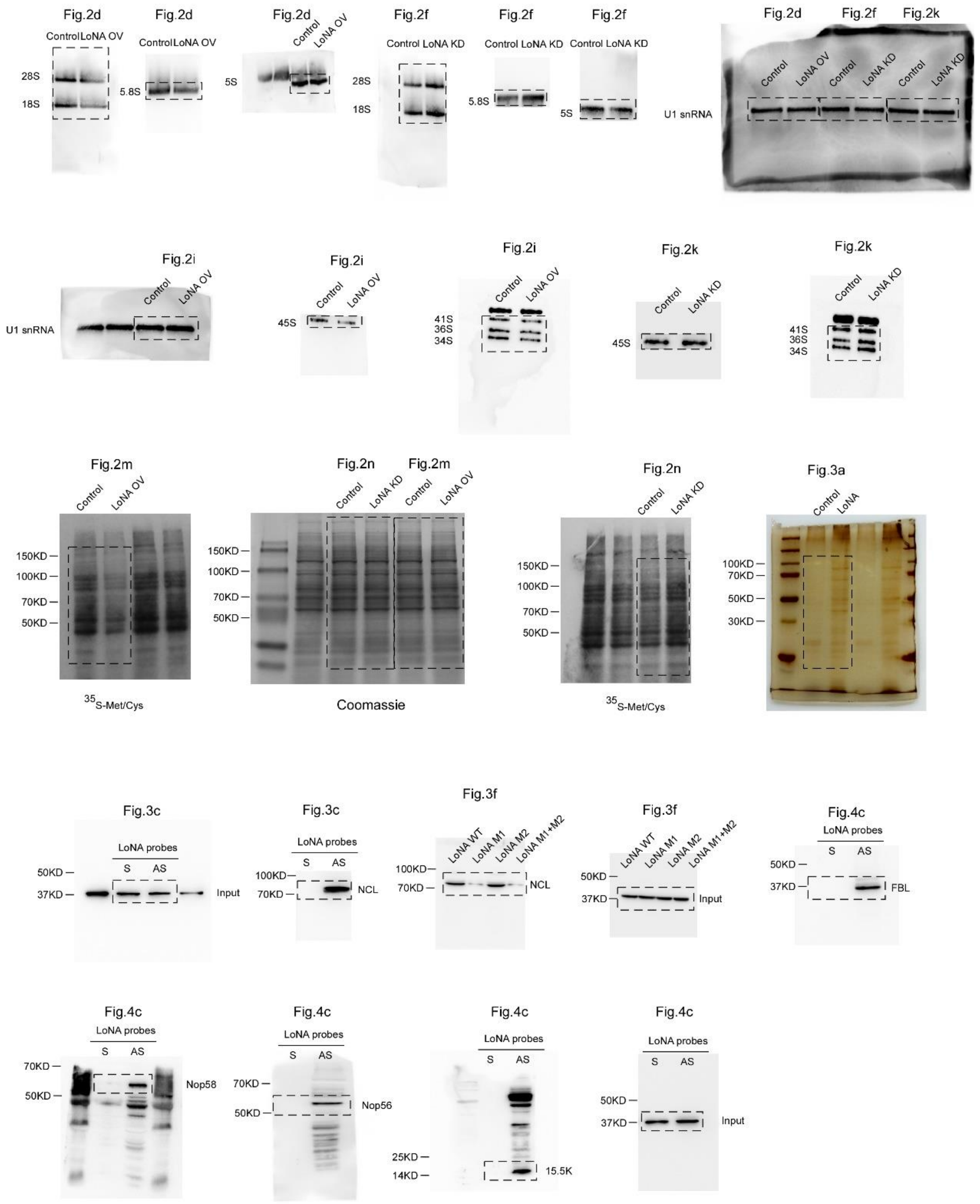

Supplementary Figure 9| Uncropped images of blots/gels from indicated figures.

Supplementary Figure 10

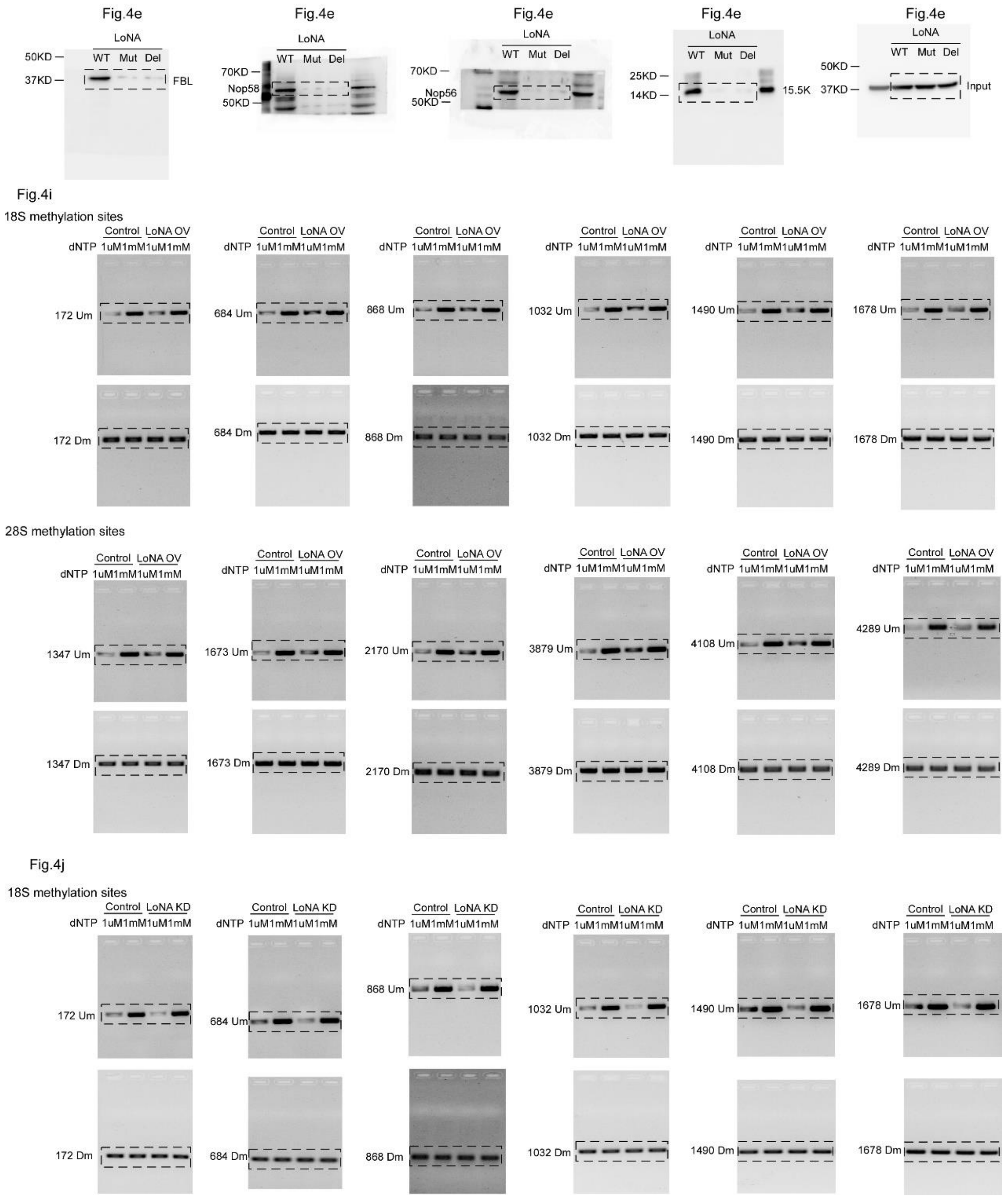

Supplementary Figure 10| Uncropped images of blots/gels from indicated figures.

Supplementary Figure 11

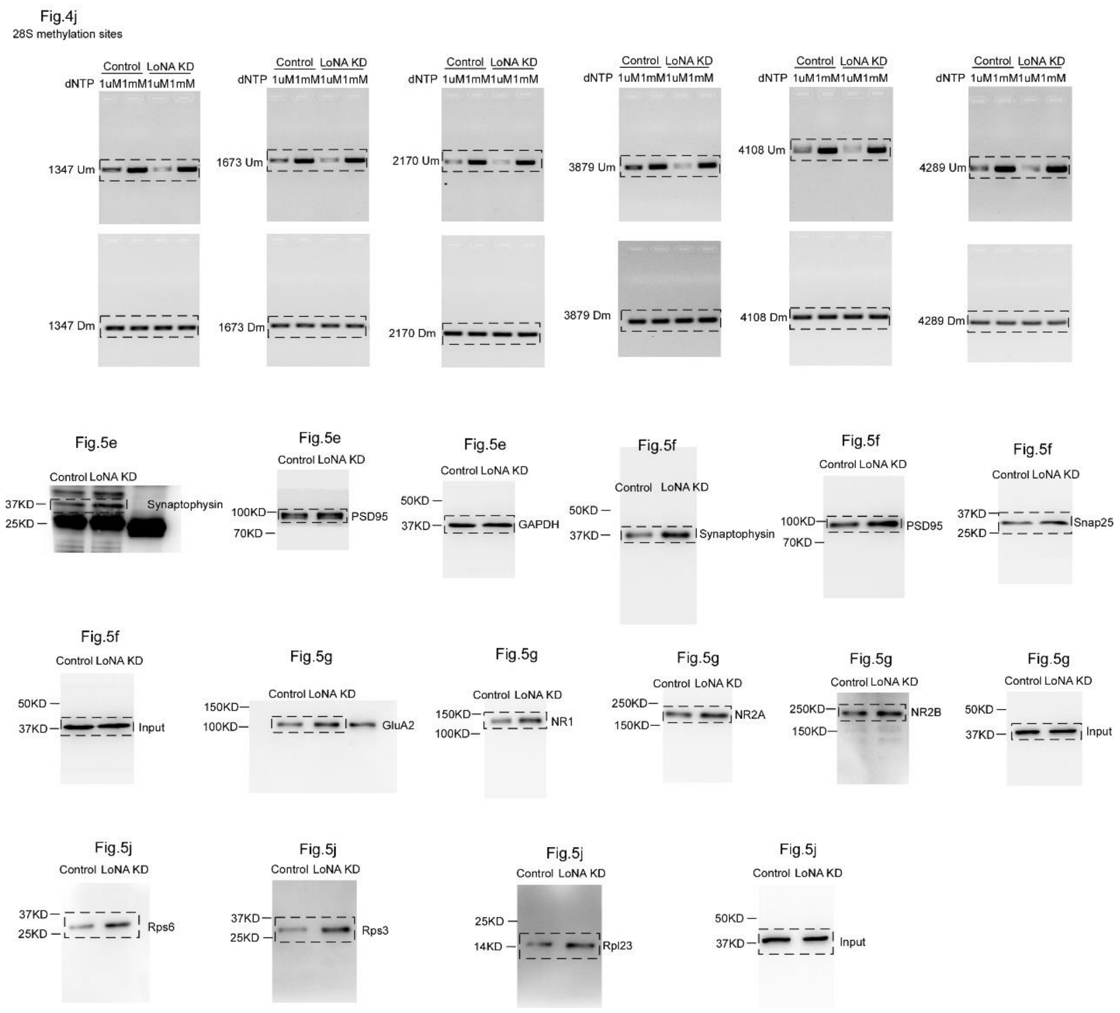

Supplementary Figure 11| Uncropped images of blots/gels from indicated figures.

Supplementary Figure 12

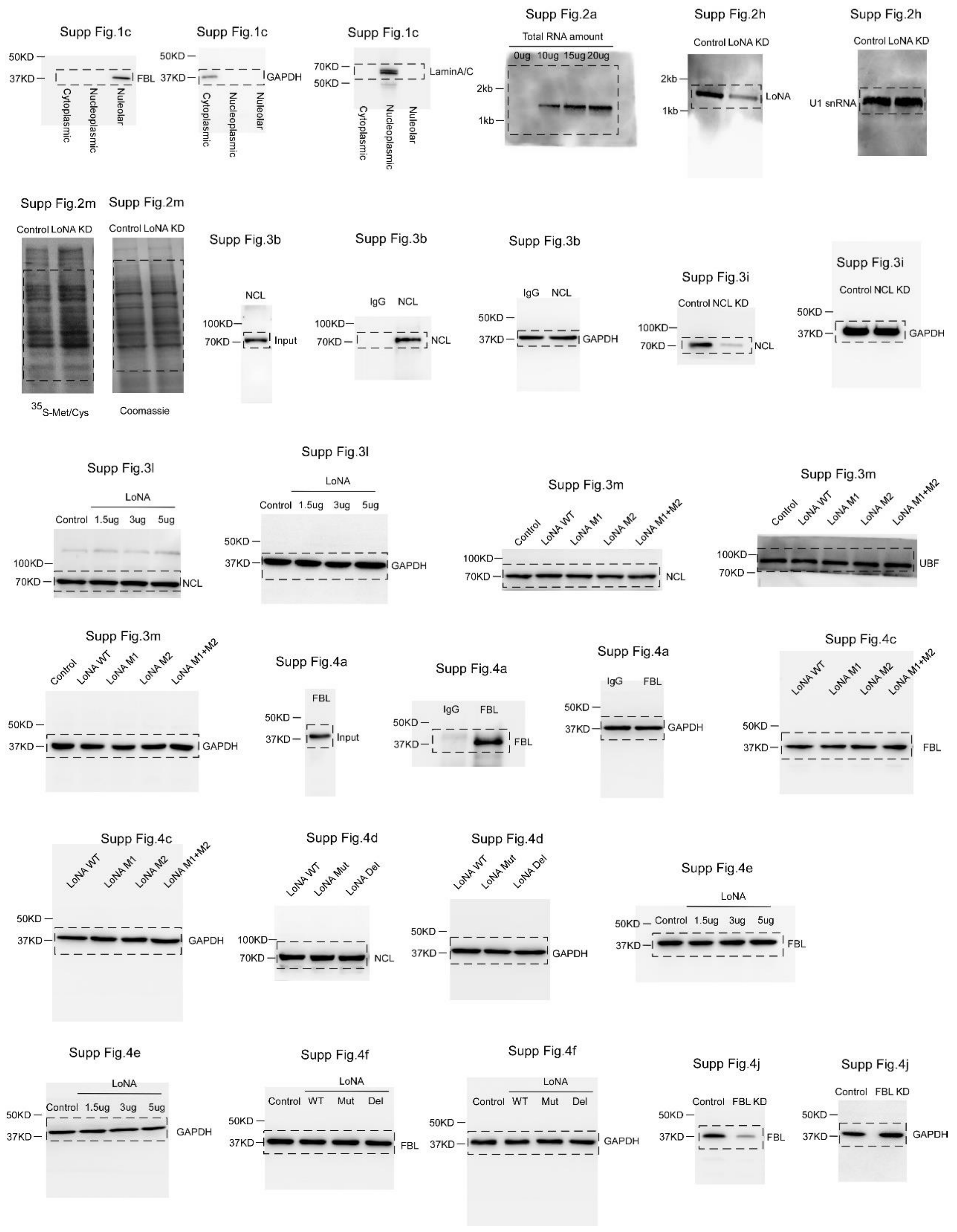

Supplementary Figure 12| Uncropped images of blots/gels from indicated figures.

Supplementary Figure 13

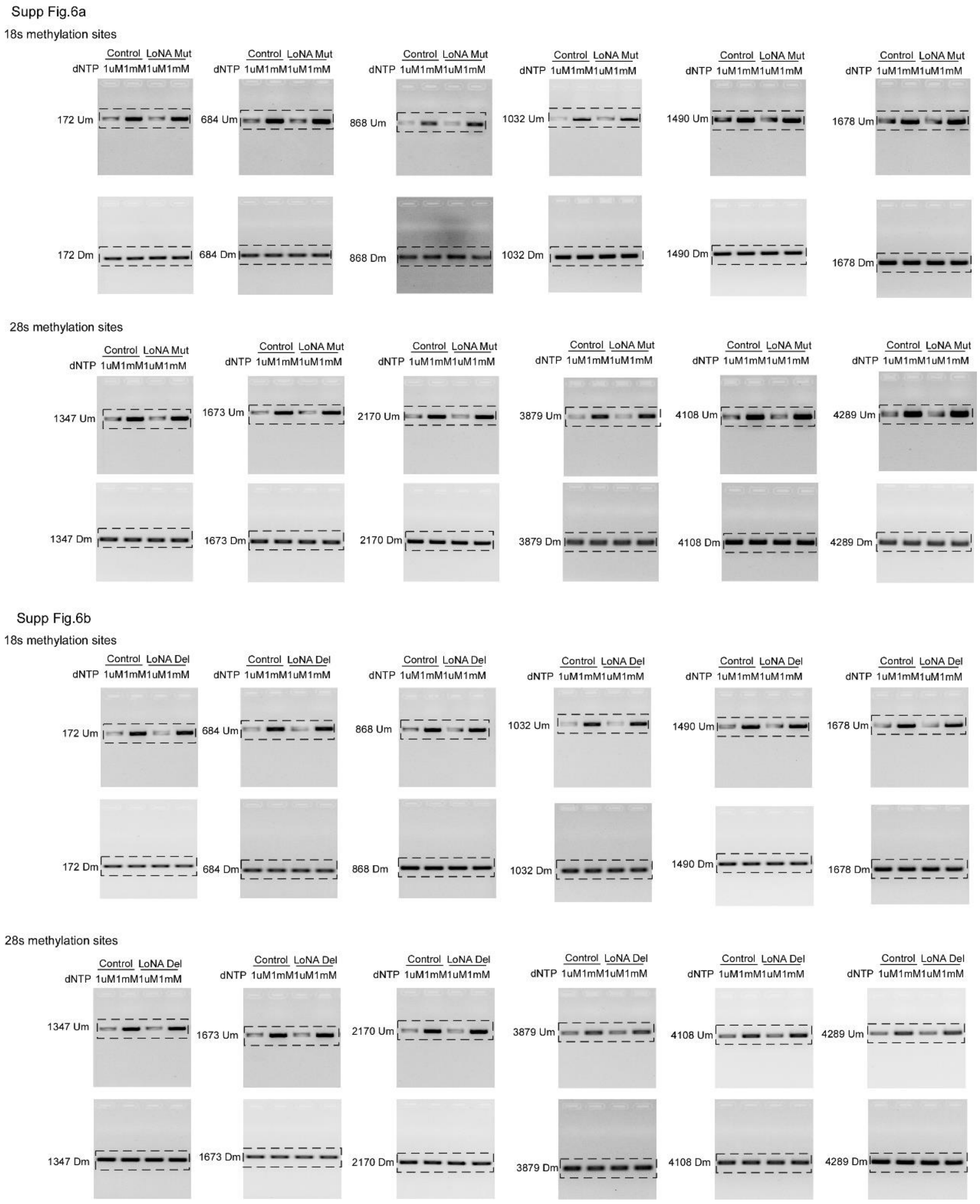

Supplementary Figure 13| Uncropped images of blots/gels from indicated figures.

Supplementary Figure 14

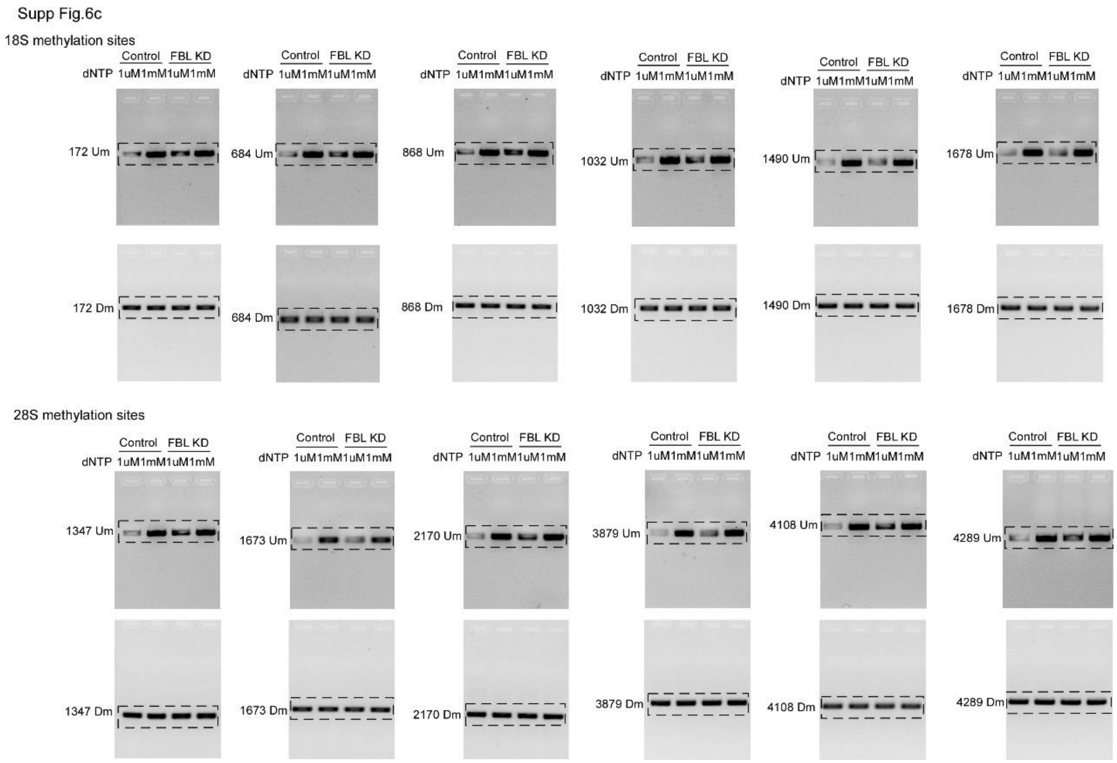

Supplementary Figure 14| Uncropped images of blots/gels from indicated figures.

Supplementary Figure 15

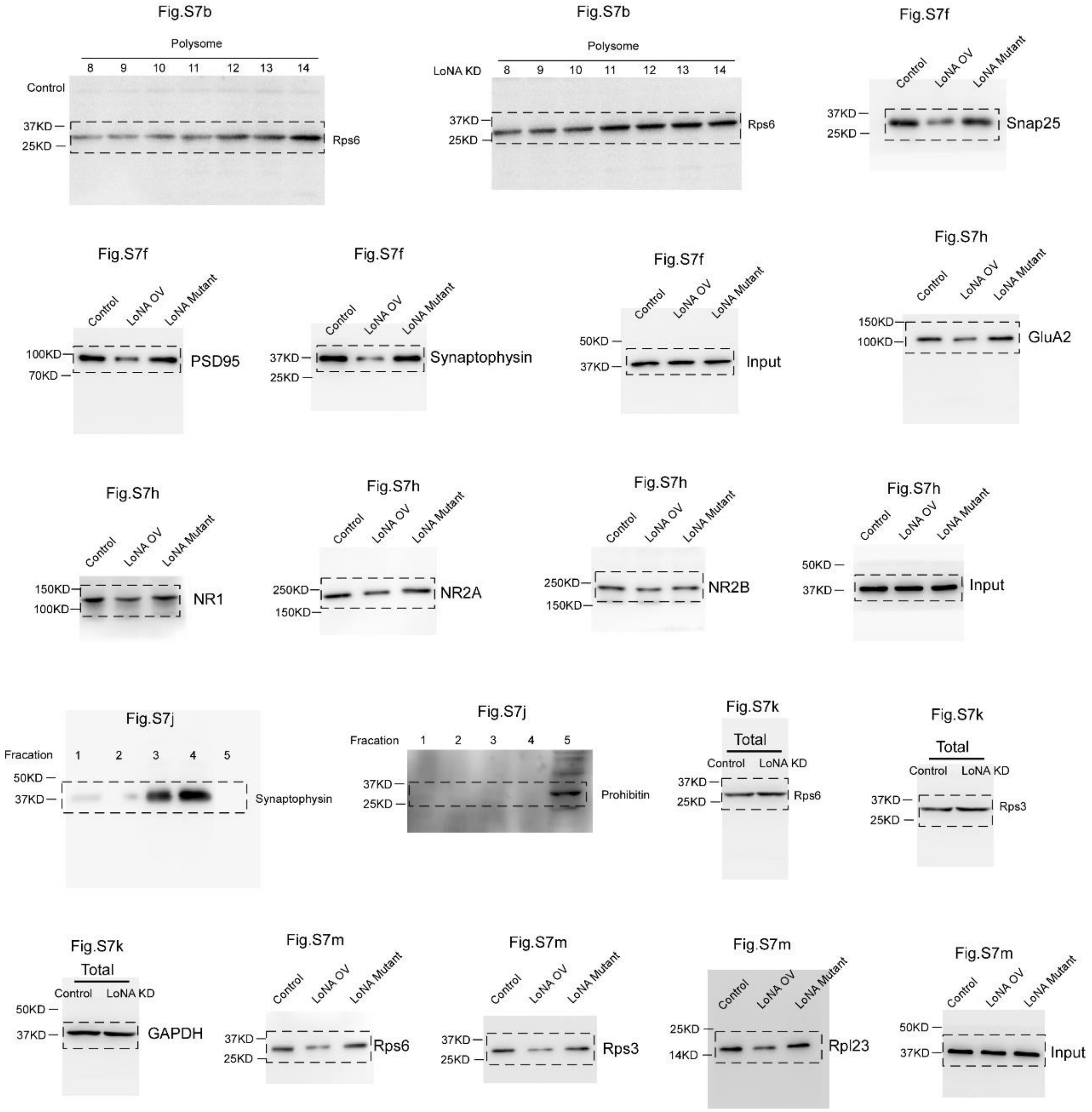

Supplementary Figure 15| Uncropped images of blots/gels from indicated figures.

Supplementary Table 1

Name of antibody	Reference	Application(s) and dilutions
Anti-GAPDH	Cat#60004 (Proteintech)	WB (1:5000)
Anti-polR1A	Cat#20595 (Proteintech)	ChIP (3μg/ChIP), WB (1:1000)
Anti-polR1E	Cat#16145 (Proteintech)	IF (1:100)
Anti-prohibitin	Cat#10787 (Proteintech)	WB (1:1000)
Anti-snap25	Cat#14903 (Proteintech)	WB (1:2000)
Anti-PSD95	Cat#20665 (Proteintech)	WB (1:2000), IP (2.5μg/IP)
Anti-synaptophysin	Cat#17785 (Proteintech)	WB (1:2000), IP (2.5μg/IP)
Anti-NOP58	Cat#14409 (Proteintech)	WB (1:1000)
Anti-NOP56	Cat#18181 (Proteintech)	WB (1:1000)
Anti-15.5K	Cat#15802 (Proteintech)	WB (1:1000)
Anti-Rpl23	Cat#16086 (Proteintech)	WB(1:800)
Anti-Rps3	Cat#11990 (Proteintech)	WB (1:800)
Anti-NCL	Cat#sc-8031 (SantaCruz Biotechnology)	WB (1:800), IP (3μg/IP), IF (1:100)
Anti-UBF	Cat#sc-13125 (SantaCruz Biotechnology)	WB (1:1000), ChIP (3μg/IP)
Anti-dimethyl H3K9	Cat#ab1220 (Abcam)	WB (1:800), ChIP (3μg/IP)
Anti-trimethyl H3K4	Cat#ab8580 (Abcam)	WB (1:800), ChIP (3μg/IP)
Anti-FBL	Cat#ab5821 (Abcam)	WB (1:3000), IP (2.5μg/IP), IF (1:500)

Anti-trimethyl H3K27	Cat#arg54767 (Arigo Biolaboratories)	WB (1:1000), ChIP (1:50)
Anti-Rps6	Cat#2317 (Cell Signaling Technology)	WB (1:1000)
Anti-His tag	Cat#2366(Cell Signaling Technology)	WB(1:1000)
Anti-GluA2	Cat#MABN1189 (Millipore)	WB(1:1000)
Anti-NR1	Cat#AB9864 (Millipore)	WB(1:1000)
Anti-NR2A	Cat#05-901R (Millipore)	WB(1:2000)
Anti-NR2B	Cat#AB1557P (Millipore)	WB(1:1000)
Anti-puromycin	Cat#MABE343 (Millipore)	WB(1:10000)
Anti-m7G/m2,2,7G	Cat#MABE419 (Millipore)	IP(4μg/IP)

WB: Western blotting; IP: Immunoprecipitation; IF: Immunofluorescence

ChIP: Chromatin immunoprecipitation

Supplementary Table 2

**RNA pull down**		**Sequence (5' to 3')**
LONA DNA sense probes	1	TAAAAGAAGTCAGTACCTGG
	2	TCTGGTAAAGTCTTCCGTCA
	3	AGTGACTACCGACCAATCTA
	4	TAAAGACTTTAGGGACACTG
	5	CTGTCAGATTCGAAGACACT
	6	CAGAGAGTCTCAACTCTCAT
	7	TGAGACTCTACGTTGTCACA
	8	GGTATATCTCCTAACTTGGG
LONA DNA antisense probes	1	ATTTTCTTCAGTCATGGACC
	2	AGACCATTTCAGAAGGCAGT
	3	TCACTGATGGCTGGTTAGAT
	4	ATTTCTGAAATCCCTGTGAC
	5	GACAGTCTAAGCTTCTGTGA
	6	GTCTCTCAGAGTTGAGAGTA

	7	ACTCTGAGATGCAACAGTGT
	8	CCATATAGAGGATTGAACCC
**LONA FISH (RNA probe, 158bp), primers for in vitro transcription**		
LONA	F	GCTAATACGACTCACTATAGGGACAGTGG GTG GCAGTTTG
	R	CAATGACCGAGGAGAACC
**Northern blot probes**		
**DNA probes**		
18S		CACCCGTGGTCACCATGGTAGGCACGGCG ACTACCATCGAAAGTTGATAG
28S		CACCTTTTCTGGGGTCTGAT
5.8S		GCAAGTGCGTTCGAAGTGT
5S		AAGTACTAACCAGGCCCGAC
U1 snRNA		CAAATTATGCAGTCGAGTTTCCCACATTTG
LoNA		ATTTCTGAAATCCCTGTGAC
**RNA probes**		
ITSI (intermediate form)		UUCUCUCACCUCACUCCAGACACCUCGCU CCACA
**Antisense oligos (ASOs)**		
LONA-ASO-1	Phosphorothioates	GACCCAAGTTAGGAGATAT
LONA-ASO-2	Phosphorothioates	GGTCGTACATAGACTTTGT
LONA-ASO-3	Phosphorothioates	GTCTCTGTGTGTGATTTAT
**LONA shRNA (vector name) target sequences highlighted RED**		
LONA-shRNA-1	top strand	GATCCCTGGGTTCAATCCTCTATACTTCCT GTCAGATATAGAGGATTGAACCCAGTTTTT G

	bottom strand	AATTCAAAAACTGGGTTCAA TCCTCTATATCTGACAGGAAGTATAGAGGA TTGAACCCAGG
LONA-shRNA-2	top strand	GATCCCCAGCATGTATCTGAAACACTTCCT GTCAGATGTTTCAGATACATGCTGGTTTTT G
	bottom strand	AATTCAAAAACCAGCATGTATCTGAAACAT CTGACAGGAAGTGTTTCAGATACATGCTGG G
LONA-shRNA-3	top strand	GATCCCAGAGACACACACTAAATACTTCCT GTCAGATATTTAGTGTGTGTCTCTGTTTTTG
	bottom strand	AATTCAAAAACAGAGACACACACTAAATAT CTGACAGGAAGTATTTAGTGTGTGTCTCTG G
**LONA box C/D mutagenesis primers**		
LONA box C/D R1	F	TCTATATGGAGAGAAAGATAGCAGATAGAT AGATAGATAGAT
LONA box C/D R1	R	ATCTATCTATCTATCTATCTGCTATCTTTCT CTCCATATAGA
LONA box C/D R2	F	ATAGATAGATTAGATAGATAGCAGATAGAT AGACAGACAGAT
LONA box C/D R2	R	ATCTGTCTGTCTATCTATCTGCTATCTATCT AATCTATCTAT
**LONA NCL binding site mutagenesis primers**		
LONA NBS R1	F	TACTTCAAGAGCAGAAGATGGAACCTTCAG ATTGGGT
LONA NBS R1	R	ACCCAATCTGAAGGTTCCATCTTCTGCTCT TGAAGTA
LONA NBS R2	F	GGACAGACATTTGAGATAATGGAGAGAGC AGTCAGTGA
LONA NBS R2	R	TCACTGACTGCTCTCTCCATTATCTCAAAT GTCTGTCC
**QPCR detection primers**		
**Ribosome RNAs**		
45S	F	GTTCCCGTGTTTTTCCGCTC
	R	AGTGCGTTCGAAGTGTCGAT
18S	F	GTAACCCGTTGAACCCCATT

	R	CCATCCAATCGGTAGTAGCG
28S	F	GTTCACCCACTAATAGGGAACGTGA
	R	GGATTCTGACTTAGAGGCGTTCAGT
5.8S	F	ACTCGGCTCGTGCGTC
	R	GCGACGCTCAGACAGG
**ncRNAs**		
LONA	F	GAGATGTCACCCACCGTCAAAC
	R	GTTACTGGCTCCTCTTGG
NCL UV-CLIP Primer	F	GCCCTATCTAACCAGCCATC
	R	TGACGGTGGGTGACAGCTA
FBL UV-CLIP Primer	F	TGGTAGAGGCAGAAGGCACT
	R	TCTCTGATCTATCTATTTATCTGTC
Malat1	F	CGTTTGAAGGCATGAGTTGG
	R	TGCCTCCCAAGTGCTAGGAT
Gm29084	F	TAAGAGCAAATAGGCCACCCATATT
	R	ATGAGCCTATTCTAGGGAGA
Gm38246	F	GTTCAATAAAACTTTGGTAAACCAG
	R	ACATCCCAACATTGAGAAAT
Gm29408	F	GTCAAAAGCACCCTCAGGTGGCCAG
	R	CGTGTCCGCTCTTGTTGTGA
Gm43549	F	GAATTTTAGAGGTCGGTGTCCCTTA
	R	CACGACCATCTTGGATAACT
Gm29562	F	CGCGCTTGGACCTGTCGCTGACCAA
	R	TGAGACTAGCCTCGTGAGAT
Gas5	F	CTTTCGGAGCTGTGCGGCATTCTGA
	R	CGCAGAACCAACCTCCTAAA
Gm26905	F	TTTTTAATGGGGTTATTTGATTTTC
	R	AATGGGACCTAATGAAACTC
Gm30948	F	TTCATATTTTAGGTTTTTTTTTTAC
	R	CCCAAGAGGCGACATATCAA
Gm26804	F	TCCCCATCAGATAATTACAGCATTT
	R	CTTCCTTGTCTACTCGTGGC
Gm42418	F	AGGGCAAGTCTGGTGCCAGC
	R	GCTCCCAAGATCCAACTACG
Gm26853	F	GCCTACTAGCTGACCTTAGACCATT
	R	GCCATTCCTGGATTGTAGTT
Gm19757	F	ATGTAAAAGAAAGCACACAAGAAAT
	R	AAATGCTGGTCTTATCTGGT
Xist	F	TGCTCCTCCGTTACATCAGACT
	R	CACTCCTCTAAATCCAGGCAATCC
Gm29055	F	ATTTAGCAGTGATAACTTGAATTGC

	R	ACTTGGGAGGTGGAAATCTA
AI5068	F	ATTGATTTGAGTTGGTGATACTGTT
	R	TCCTCGGACCCTGCATTCTC
Gm42428	F	CTCAGCCTTTTTAGAGAGACTCAAA
	R	CAGACTGCCAGATGAATGAC
Gm43190	F	TCATATGTCAAAATTACAGTTCATC
	R	TAGATATGACACTGCCCACT
O14Rik	F	GGGGTGTCAATCGGAGGAAAATCTC
	R	CTTCCCTATTAAACACCCAC
Gm16344	F	ACCACCTTGGTCAGTCCTTTCCAAC
	R	AGTCAGTTGCCTCATTCTAC
Gm43478	F	GAGGGAGAGAACACCCAGACTGCAG
	R	CTCTGGAGCCAGGGTACTTG
Gm28268	F	GGTTCTGCTTGGAGGGTGGCATAGA
	R	GACTGGAGGCTTTGTTTGAT
Gm37724	F	ATAGTTTCTTCCTTCAAGAGGGAAA
	R	TTTCCCTCTTGAAGGAAGAAACTAT
Gm19757	F	TAAAATTTATTTACCAAGCCATGTG
	R	AAATGCTGGTCTTATCTGGT
Gm20186	F	ACGGGATGGGAGGGACAGGGGAATG
	R	CTGCCTCCCACTCCAACTCC
Gm14149	F	GCGCCGCCTGCTTTCCTGTCACCAG
	R	TATAGCAGGGTGGAATAGAG
J07Rik	F	CCTTCTGCTCGACTCGAGCCCCGGG
	R	CCCTATGTACCGCAGTCTCA
Gm15758	F	GAGATATAAAAACAGGGGAGATATA
	R	AACAGGCCAGGAAGAACGAC
Gm26869	F	CCTTCCTGAGTGTGGATTAATGGGG
	R	TTGTATTTGCTGGAACCCTC
U1 snRNA	F	GGGAGATACCATGATCACGAAGGT
	R	CCACAAATTATGCAGTCGAGTTTCCC
U3 snoRNA	F	CTGACTGTGTACAGCACCCG
	R	TCCACTCAGACCTGCGTTCCC
**Ribosomal RNA chromatin**		
UCE	F	CTCCCGCTCTGGAGACAC
	R	GGACACCTGTCCCCAAAAAC
CORE	F	AGGTGTCCGTGTCCGTGT
	R	GCCCAAAATTGCCGACTC
18S coding region	F	CGACGACCCATTCGAACGTCT
	R	CTCTCCGGAATCGAACCCTGA

18S coding 5' end	F	GGATGCGTGCATTTATCAGA
	R	GATCGGCCCGAGGTTATCTA
28S coding region	F	AGTCGGGTTGCTTGGGAATGC
	R	CCCTTACGGTACTTGTTGACT
28S coding 3' end	F	ACCTGGCGCTAAACCATTCGT
	R	GGACAAACCCTTGTGTCGAGG
**Ribosomal RNA methylation sites**		
18S (172)	Fu	CTGTGGTAATTCTAGAGCTAA
	Fd	GGATGCGTGCATTTATCAGA
	R	TGATAGGGCAGACGTTCGA
18S (684)	Fu	GCAGTTAAAAAGCTCGTAGTT
	Fd	GAGGCGAGTCACCGCC
	R	GCGGGACACTCAGCTAAG
18S (868)	Fu	GATACCGCAGCTAGGAATAAT
	Fd	CATGATTAAGAGGGACGG
	R	CTTGATTAATGAAAACATTCTT
18S (1032)	Fu	CTTGATTAATGAAAACATTCTT
	Fd	ACCATAAACGATGCCGACTG
	R	CCGGAACCCAAAGACTTTG
18S (1490)	Fu	CACCCGAGATTGAGCAATAACA
	Fd	TACACTGACTGGCTCAGCGTG
	R	GCTTATGACCCGCACTTA
18S (1678)	Fu	GGTCATAAGCTTGCGTTGATT
	Fd	GGTTTAGTGAGGCCCTCG
	R	TCCTTCCGCAGGTTCACC
28S (1347)	Fu	GGACCCGAAAGATGGTGA
	Fd	CGGTCCTGACGTGCAAAT
	R	GTCGGGAGCGAGAGCGC
28S (1673)	Fu	TGGGCCACTTTTGGTAAGC
	Fd	CGCTCATCAGACCCCAG
	R	ACGCTCCAGCGCCATCC
28S (2170)	Fu	AGTGGAGAAGGGTTCCATG
	Fd	GAACAGCAGTTGAACATGG
	R	GGATCGGTCGCGTTACC
28S (3879)	Fu	GCTCCGGGGACAGTGC
	Fd	GTCCTAAGGCGAGCTCAG
	R	TGAAAATCAAGATCAAGCGAG
28S (4108)	Fu	GCGTTCATAGCGACGTCG
	Fd	CTTCCTATCATTGTGAAGCA

	R	GAACCTGCGGTTCCTCTC
28S (4289)	Fu	AGGTTCAGACATTTGGTGTAT
	Fd	GCTTGGCTGAGGAGCCAA
	R	CTATCCGGGGCCAACCG
**Histone methylation in promoter region of rRNA chromatin**		
H3K4me3	F	GACACAGGAGAGGGAAGTGC
	R	CTCCCTGTACGACCTCCTTG
H3K9me2	F	GACCAGTTGTTCCTTTGAGG
	R	ACCTATCTCCAGGTCCAATAG
H3K27me3	F	CTTGCGTGTGCTTGCTGT
	R	GAAATCGGGAAAAACGTCTG
**Histone methylation in coding region of rRNA chromatin**		
H3K4me3	F	CATCTGCTCTGGTCGAGGTT
	R	GCAAGACCCAAACACACACA
H3K9me2	F	ACTGACACGCTGTCCTTTCC
	R	GACAGCTTCAGGCACCGCGA
H3K27me3	F	ACACCCGAAATACCGATACG
	R	AGTGCGTTCGAAGTGTCGAT
**Genes**		
GAPDH (mouse)	F	AGGTCGGTGTGAACGGATTTG
	R	TGTAGACCATGTAGTTGAGGTCA
GAPDH (human)	F	AGGGCTGCTTTTAACTCTGGT
	R	CCCCACTTGATTTTGGAGGGA
FBL	F	CAAAATTGAGTACAGAGCCTGGA
	R	CGGGCCGACAATATCAGAGA
PSD95	F	GGCGGAGAGGAACTTGTCC
	R	AGAATTGGCCTTGAGGGAGGA
Synaptophysin	F	CAGCACAACATACCCTGTGG
	R	GGTCTTCCAGTTACCCGACA
NCL	F	AAAGGCAAAAAGGCTACCACA
	R	GGAATGACTTTGGCTGGTGTAA

Supplementary Methods

Nucleoli isolation and High-Throughput Sequencing

N2a cell nucleoli were isolated as previously described[1]. Briefly, cells were collected by trypsinization and resuspended in buffer containing 10 mM HEPES pH 7.9, 10 mM KCl, 1.5 mM $MgCl_2$ and 0.5 mM DTT. Cell suspensions were then subjected to homogenization and centrifugation. Pellet (nuclear fraction) was re-suspended in buffer containing 0.25 M sucrose and 10 mM $MgCl_2$, followed by sonication. Sonicated samples were subjected to sucrose gradient centrifugation, the pellet (nucleoli fraction) was saved for further analyses.

Total RNA from nucleoli was extracted by Trizol (Invitrogen), ribosomal RNA was removed using Ribo-Zero Gold LT Sample Prep Kit (Illumina) and RNA-seq libraries were prepared using the TruSeq RNA Kit (Illumina) according to the manufacturer's protocol and subject to Illumina HiSeq 2500 platorm for 150 bp pair-end sequencing. The low quality sequenced reads ($Q \leq 10$) were removed and clean reads were aligned on Enseml (version mm10) and GENCODE (version 19) of the mouse genome, using TopHat.

Plasmids construction, ASO and transfection

All plasmids were constructed with restriction-enzyme digestion (NEB) and ligation methods (Vazyme). LoNA overexpression vector was generated by inserting its sequence into SnoVector[2] for nuclear specific expression. Three different LoNA shRNA sequences were cloned into pGreenPuro hairpin (H1) backbone lentivector (SBI) separately. H1 promoter and LoNA shRNA sequences were amplified from SBI vector and cloned into pAAV-EF1a-DIO-Gcamp6s vector (Addgenes) for AAV virus production, EF1a promoter was replaced with CamkIIa promoter followed by zsGreen sequence for neuronal specific visualization of injected area. Correct insertions were confirmed by gel electrophoresis and DNA sequencing. The shRNA plasmid for knockdown of NCL mRNA (TRCN0000071172), FBL mRNA (TRCN0000097576)

along with negative control shRNA (SHC002) were obtained from the MISSION shRNA Library (Sigma). Phosphorothioate antisense oligonucleotides (ASOs), with nucleotides at the 5’- and 3’- ends replaced with phosphorothioate oligonucleotides, were synthesized by General Biosystems. ASO transfection was performed with Lipofectamine RNAi Max (Invitrogen) and other plasmids transfection was conducted with Lipofectamine 2000 (Invitrogen) according to manufacturer’s protocol.

Generation of lentivirus

Lentivirus plasmid was transfected into 293T cells together with packaging plasmid (pHR’8.2deltaR) and envelope plasmid (pCMV-VSV-G), virus containing medium was harvested 24 h and subjected to ultracentrifuge for virus precipitations. The virus was resuspended in PBS and viral titer was determined by qPCR.

Luciferase vectors and assays

45S pre-rRNA promoter (GenBank: BK000964.3) was cloned into PNL2.1 construct (Promega) according to manufacturer’s protocol[3]. To assess if LoNA alters 45S promoter activity, N2a cells were transfected with LoNA, mutant LoNA, LoNA antisense or NCL-shRNA (positive control). 24 h after the initial transfection, 45S pre-rRNA promoter luciferase vector was introduced into these cells. Cells were lysed 24 h later and lysates were incubated with luciferase substrate. Data were presented as luciferase activities.

Quantification of RNA copy number per cell

DNA fragments corresponding to LoNA were amplified from mouse cDNA, and purified fragments were used to plot standard curves by qPCR. Total RNA was extracted from $1.0x10^5$ cells, followed by cDNA synthesis. The copy numbers per cell in each cell line were calculated on the basis of cell numbers and the Ct value by using the standard curve.

m^7-GTP/TMG ($m^{2,2,7}$G) and oligo(dT) Pull-Down Assays

RNA extracted from nuclei was incubated with agarose beads conjugated with m^7-GTP/$m^{2,2,7}$-Trimethylguanosine antibody (Millipore) or control IgG, followed by qPCR analyses for antibody bound RNA. U1 snRNA, U3 snoRNA and FBL were included as positive controls.

RNA was incubated with Dynabeads® Oligo (dT) 25 (Invitrogen) for 2 h at 4°C to isolate either poly(A)+ RNAs, which were bound to beads, or poly(A)- RNAs, which were present in the flow through after incubation. The poly(A)- RNA samples were subjected to rRNA removal (RiboMinus kit, invitrogen), followed by RNA precipitation and purifications. Poly (A)+ RNA bounded beads were centrifuged at 1,000 g for 5 min, followed by three times PBS wash, then subjected to RNA extraction and qPCR analyses. The levels in poly (A)- fractions were normalized against levels in poly (A)+ fractions for GAPDH and LoNA, and levels in poly (A)+ fractions was normalized against levels in poly (A)- fractions for U1 snRNA.

Silver staining and Mass-Spectrometry

Silver staining was performed according to a previous published protocol[4]. RNA pulldown protein samples were resolved on electrophoresis gels, then were placed in fixing solution (50% ethanol, 12% acetic acid and 0.05% formalin) for 1 h and washed with 20% ethanol for 20 min. Gels were subsequently sensitized with 0.02% sodium thiosulfate and stained with cold 0.2% silver nitrate for 20 min. Silver stained gels were developed using a solution containing 6% sodium carbonate, 0.0004% sodium thiosulfate and 0.05% formalin. The reaction was terminated with 12% acetic acid. Bands were cut, digested, extracted from gels and subjected to mass-spectrometry analyses (PTM Biolabs).

Pulldown of biotinylated RNA

Pulldown of biotinylated RNA was carried out according to published previously[5], with modifications. Full length WT LoNA and its variants were *in vitro* transcribed and biotin-labeled with Biotin RNA labeling kit (Epicentre). Biotinylated RNAs were then treated with RNase-free DNaseI (Promega) and extracted with

phenol/chloroform/isoamyl alcohol. N2a cell were lysed with RIP buffer (150 mM KCl，25 mM Tris pH 7.4, 5 mM EDTA, 0.5 mM DTT, 0.5% NP-40, cocktail proteinase inhibitor (Roche)) for 30 min at 4°C, then lysates were subjected to sonication for 5 min. Cell debris were removed by centrifugation at 14,000 r.p.m. for 10 min at 4°C, protein concentrations of supernatants were determined by BCA protein assay kit (Pierce). Biotinylated RNA probes were heated at 60°C for 40 min and slowly-cooled over a course of 40 min to 4°C. Equal amount of cell lysates were incubated with biotin labeled RNA probes for 2 h at 4°C in present of RNase inhibitor, 5 mM $MgCl_2$ and 0.1 mg ml^{-1} salmon sperm DNA, followed by incubation with M-280 streptavidin magnetic Dynabeads (Invitrogen) for 2 h at 4°C. Beads were captured by magnets (Life Technologies) and proteins were eluted for Western blot analysis.

Puromycin Incorporation Assay (SUnSET assay)

SUnSET was performed according to previously published protocol[6], with modifications. Briefly, N2a cells were transduced with LoNA or LoNA shRNA along with controls, and 72 h later were pulsed with 10 μg ml^{-1} puromycin for 1 h. Cells were then lysed in buffer (150 mM KCl, 25 mM Tris pH 7.4, 5 mM EDTA, 0.5 mM DTT, 1% NP40, 100 U ml^{-1} RNAase inhibitor, cocktail Protease inhibitors (Roche)) and protein concentration was determined by BCA protein assay kit (Pierce). Equal amounts of lysate were subjected to immunoprecipitation with anti-synaptophysin or anti-PSD95 antibodies, followed by immunoblotting with anti-puromycin antibody.

^{35}S Incorporation Assays

Cells were washed twice with methionine/cysteine-free media (Life technologies) and then incubated with Met/Cys-free media containing 10% fetal bovine serum, and 100 μCi ml^{-1} EasyTag EXPRESS ^{35}S Protein Labeling Mix for 30 min. Cells were washed twice with ice cold PBS containing 100 μg mL^{-1} cycloheximide, then lysed with RIPA buffer. Extracted proteins were subjected to protein concentration determination by BCA kit (Pierce) and separation on 10% SDS-PAGE. Gels were dried for two

hours at 80°C and exposed 24 h on a phosphorimager screen at -80°C freezer. Signal was quantified by ImageJ software and normalized to total protein determined by Coomassie staining.

Polysomal profiling assays

N2a cells were pre-treated with cycloheximide (Sigma) for 15 min at 37°C, and harvested in lysis buffer (50 mM Tris-HCl, 5 mM $MgCl_2$, 100 mM KCl, 1%NP-40, 1% Sodium Deoxycholate, proteinase inhibitor, 40U μl^{-1} RNase inhibitor). Nuclei and mitochondria were removed by centrifugation at 14,000 rpm for 10 min at 4 °C. Ribosomal particles (60S large subunit, 80S monosome, and polysome) were then separated on 10-50% sucrose gradient by ultracentrifugation at 38,000 rpm for 2.5 h at 4°C. Ribosome distribution was analyzed by measuring the absorbance of each fraction at OD 260 nm (A260) by UV photometry. Sucrose gradient was prepared using Gradient Master™ (Biocomp) and fractionation was Piston Gradient Fractionator™ (Biocomp). RNA from each fraction was isolated with TRIzol (Invitrogen) according to the manufacturer's instructions.

Chromatin Immunoprecipitation (ChIP)

ChIP assays were performed as described previously[7], with modifications. Briefly, cells were trypsinized and collected in PBS containing 1% formaldehyde (vol/vol) for 10 min at RT to cross-link proteins to chromatins. Cross-linking was then quenched with 125 mM glycine for 5 min. Cross-linked cells were then pelleted by centrifugation at 800 *g*, and resuspended in lysis buffer (50 mM HEPES-KOH pH 7.5, 140 mM NaCl, 1 mM EDTA pH 8.0, 1% Triton X-100, 0.1% Sodium Deoxycholate, 0.1% SDS, Complete proteinase inhibitor (Roche)). Cell extracts were sonicated with SCIENTZ-IID (SCIENTZ) to shear DNAs to a size range of 200 to 500 bp, cell debris were removed by centrifugation at 8000 *g*. A 50 µl of supernatant was saved as an input, and the remaining was pre-cleared using protein A/G magnetic beads, then diluted 10-fold in dilution buffer (50 mM Tris-HCl pH 8.0, 150 mM NaCl, 2 mM EDTA pH 8.0, 1% NP-40, 0.5% Sodium Deoxycholate, 0.1% SDS, cocktail Protease

Inhibitors (Roche)). Diluted samples were incubated with protein A/G magnetic beads conjugated with target antibodies or control IgG (Santa Cruz) at 4°C overnight. The beads were then subjected to low salt buffer wash (0.1% SDS, 1% Triton X-100, 2 mM EDTA, 20 mM Tris-HCl pH 8.0, 150 mM NaCl), high salt buffer wash (0.1% SDS, 1% Triton X-100, 2 mM EDTA, 20 mM Tris-HCl pH 8.0, 500 mM NaCl) and LiCl buffer wash (0.25 M LiCl, 1% NP-40, 1% sodium deoxycholate, 1mM EDTA, 10 mM Tris-HCl pH 8.0) to remove non-specific bindings. The antibody/protein/DNA complexes were eluted (1% SDS, 100 mM $NaHCO_3$) and the DNA/protein cross-links were reversed for 3 h at 65°C. Samples were digested with 2 μg μl^{-1} proteinase K (Merck) and 0.02 μg μl^{-1} RNase A (Vazyme). DNA was purified using PCR purification kit (Sangon Biotech) and used as template for quantitative PCR. For histone methylation analyses, target antibodies were anti-dimethyl H3K9 or anti-trimethyl H3K4 or H3K27. For UBF or PolI ChIP, target antibodies were UBF or polI1A. Primers to detect different chromatin regions are listed in Supplementary Table 1.

UV Cross-linking and Immunoprecipitation (UV-CLIP) Assay

N2a cells were cross linked with 0.4 J cm^{-2} of 254 nm UV light (UV-CLIP) and then lysed with Cell Lysis Buffer (Cell Signaling Technology) containing 1U μl^{-1} RNase (Vazyme) and proteinase inhibitors (Roche). Equal amounts of cell lysate were incubated with protein A/G beads (Invitrogen) pre-bounded with anti-FBL or NCL antibody along with control IgG (Proteintech). Beads were subjected to High salt wash buffer (50 mM Tris-HCl pH 7.4, 1 M NaCl, 1 mM EDTA, 1% NP-40, 0.1% SDS, 0.5% sodium deoxycholate) for three times, followed by wash buffer (20 mM Tris-HCl pH 7.4, 10 mM $MgCl_2$, 0.2% Tween-20) for another three times. Beads bounded total RNA was extracted by Trizol (Invitrogen) and subjected to qPCR analysis. Primers to for detection are listed in Supplementary Table 1.

rRNA methylation site determination

rRNA methylation was determined by RTL-P method according to published protocol[8]. Site-specific rRNA methylation levels were quantified at 12 different sites, selected in 18S and 28S ribosomal RNA respectively. For the detection of 2'-O-methylated sites in the target rRNAs, site-specific primer extension by reverse transcriptase was performed with low or high concentration of dNTP respectively, followed by semi-quantitative PCR amplifications. Reverse transcription of rRNA into cDNA were carried out using reverse primers targeting a sequence upstream to a specific methylation site, in presence of either a low level (1μM) or a high level (1mM) of deoxynucleoside triphosphate (dNTP). RT reaction mixture contained 20 ng of total RNA, 200 U SuperScript II Reverse Transcriptase (Invitrogen), 10 mM specific reverse primers and 1 μM (low) or 1 mM (high) dNTPs (Vazyme). The cDNA was then amplified by two separate PCR reactions with two pairs of specific primers targeting upstream and downstream of a specific methylation site respectively. The PCR reaction mixture contained 1μl of the cDNA template, 1.25 U DNA Tag polymerase (Vazyme), 10 mM dNTP, and 10 mM of site specific forward and reverse primers. The PCR products were then separated on 2% agarose gels, stained with Ethidium Bromide (Sigma) and visualized by UV trans-illumination. PCR signal intensities were analyzed using ImageJ software. The methylation ratio of each site was determined from density of PCR bands obtained using high and low dNTP concentrations.

Generation of Adeno-associated virus and hippocampal injection

Adeno-associated virus (AAV) was produced according the protocol described by Guo et al[9]. Briefly, target plasmid, pHelper and helper2/9 were co-transfected into 293T cells in a ratio of 2:1:1. PEI (Sigma) was used as transfection reagent with ratio of PEI:total DNA maintained at 4:1 (weight/weight). 24 h after transfection, medium was changed to DMEM+2%FBS. 72 h after transfection, cells were pooled and lysed in buffer containing 50 mM Tris-HCl, 150 mM NaCl, 2 mM $MgCl_2$, pH 8.0, AAV particles were released with three freeze/thaw cycles, followed by 50 U ml^{-1} benzonase (Seven Sea) and 10 U ml^{-1} RNase I (Vazyme) incubation at 37°C for 30

min, and 0.5% sodium deoxycholate (Sigma) for another 30 min. 2,500 *g* centrifugation for 10 min to get AAV containing supernatant, and 40% PEG8000 together with 2.5 M NaCl was added to precipitate the virus. Pellet was resuspended in PBS, followed by chloroform and $(NH4)_2SO_4$ treatment to remove contaminated proteins.

Male WT C57BL/6J mice at 10 weeks of age (n=10 each group) were stereotaxically injected with adenovirus expressing either LoNA-shRNA (0.5 µl, $4x10^{11}$ TU ml^{-1}) or control AAV (0.5 µl, $8x10^{11}$ TU ml^{-1}) into the bilateral CA1 area of hippocampus with an air pressure injector system (KDS). The coordinates used for stereotaxic injections was AP -2.3, ML 2.0, DV -1.5 and AP -2.3, ML -2.0, DV -1.5. Behavioral tests were performed 21 days after the injection.

Behavioral tests

The Morris water maze was performed according to a previously published protocol[10]. Briefly, mice (n=10 each group) were trained in the Morris water task to navigate a direct path to the hidden escape platform when started from semi-random locations around the perimeter of the tank. Four training trials were given to each mouse per day for 5 consecutive days during the learning phase. After total 20 training trials, each mouse was given a 30 second probe trial with the platform removed. Time spent in the target quadrant when compared with other quadrants and how often the mice entering the target quadrant was determined. Mice were sacrificed 24 h after training and tissues were saved for RNA analysis. This time window was kept consistent between experiments.

The object-context discrimination test was performed as described[11]. Briefly, mice were placed in an open chamber with a specific floor pattern and two identical objects, followed by 10 min exploration and an inter-trail interval. Mice then were placed in a second chamber with different floor pattern and the objects unique from the objects in the first trial. Mice finally were tested for 10 min in a chamber consisting of a floor pattern from either trial one or trial two, one object from trial

one, and one object from trial two. The time mice spent in exploring the object in novel context was compared to the different object in the old context. Mice were sacrificed 24 h after training and tissues were saved for RNA analysis. This time window was kept consistent between experiments.

The fear conditioning test was carried out as described previously[12]. Briefly, on day 1, mice were allowed to explore the chamber for 3 min and then exposed to 20 sec tone (85 dB, 2700 Hz) (conditioned stimulus), after a trace period of 20 sec, a mild foot shock (2 sec, 0.5 mA) (unconditioned stimulus) was administered to the mice. 5 conditioning trials (pairing) were performed with a 200 sec inter-trial interval. On day 2, trace memory was evaluated. A 2 min baseline period followed by three 20 sec tone with 220 sec interval was presented to mice, and freezing behavior and time was recorded. On day 3, contextual memory was assessed. The context should be identical to that of day 1. Freezing behavior was recorded for 8 min. Mice were sacrificed 24 h after training and tissues were saved for further analysis. This time window was kept consistent between experiments.

Synaptosomes purification

Synaptosomes (n=7 each group) were isolated using discontinuous Percoll (GE Healthcare) gradients as described previously[13]. Briefly, hippocampal brains were homogenized in sucrose buffer (0.32 M sucrose, 1 mM EDTA, 5 mM Tris pH 7.4, 0.25 mM DTT) containing cocktail protease inhibitor (Roche) and centrifuged at 3,600 *g* at 4 °C. Supernatant was layered on top of a discontinuous Percoll gradient (3-23%, Percoll; 0.32 M sucrose, 1 mM EDTA, 5 mM Tris pH 7.4, 0.25 mM DTT) and subjected to centrifugation at 31,000 *g*. Fractions 3-5 were collected, diluted with sucrose buffer and centrifuged at 29,000 g. Pellets were synaptosomes and saved for further analyses.

Electrophysiology

C57BL/6J mice were administered with LoNA or LoNA shRNA in hippocampal brain through adeno-associated virus delivering system (n=10 for each group). Three

weeks after the injection, mice were anaesthetized and placed in the stereotaxic apparatus. The recording electrode was placed at the cell body layer of CA1 and the stimulating electrode at the CA3 according to stereotaxic parameters. The long-term potentiation (LTP) was then evoked and recorded. The parameters for theta burst stimulus to induce LTP were five trains of 20 pulses at 200 Hz stimulation for 1 s with each train separated by a 1 min interval.

Golgi Staining and Dendritic Spine Analysis

Golgi staining was performed as described previously[14]. Briefly, freshly dissected brains were immersed in Golgi-Cox solution (5% w/v $K_2Cr_2O_7$, 5% w/v $HgCl_2$, 5% K_2CrO_4) for 2 weeks at room temperature and then transferred to Tissue Protection solution (0.3% w/v NaH_2PO_4, 1% w/v Na_2HPO_4, 1.8% w/v NaCl in 100 mM PBS) for 24 h at RT. Brain sections were cut at 150 µm using Leica vt1200 microtome, then mounted onto slides for visualizations. The pyramidal neurons in the CA1 region of the hippocampus were visualized and analyzed. Five neurons were randomly selected per brain region from each mouse, and at least two segments were chosen randomly per neuron. Dendritic spines density was measured in a blinded manner.

Western Blot and Densitometry Analysis

Cells or brain tissues were lysed on ice in RIPA buffer (50 mM Tris pH 8.0, 150 mM NaCl, 1% Triton X-100, 0.5% sodium deoxycholate, 0.1% SDS). Protein concentrations were determined using a BCA Protein Assay kit (Pierce). Equal amounts of protein were used for SDS-PAGE. The immunoreactive bands were visualized by enhanced chemiluminescence (Thermo Scientific) and detected by ChemiScope (CLiNX). For densitometric analyses, immunoreactive bands were quantified using Image J software.

RNA extraction, Reverse transcription and Quantitative PCR

Total RNA was isolated from cells or tissues using Trizol (Invitrogen) and subjected to DNase I digestion to remove genomic DNA contamination. Total RNA was

dissolved in nuclease-free water and stored at -80 °C for further use. Reverse transcription was performed using SuperScript II RNase H-reverse transcriptase (Invitrogen) following the manufacture's instruction. Quantitative PCR was performed with SYBR Green qPCR Master Mix (Roche) on LightCycler 96 system (Roche) according to standard procedures. The real-time value for each sample was averaged and compared using the CT method, where the amount of target RNA ($2^{-\Delta\Delta CT}$) was normalized to an endogenous reference (ΔCT) and related to the amount of target gene in tissues or cells, which was set as the calibrator at 1.0. QPCR detection primers are listed in Supplementary Table1.

Primary Neuronal Culture

Mouse primary neurons were prepared from embryonic day 17 of C57BL/6J mice as previously described[15]. Neurons were seeded at a density of 500,000 cells per 6cm dish or 350,000 per 6 well plate. Cultures were grown in Neurobasal medium supplemented with B27 (Invitrogen).

Confocal Microscopy

Fluorescence signals were captured with HRm CCD camera mounted on LSM710 confocal microscope system (Zeiss), with ZEN software.

Statistical Analysis

All quantified data represent an average of at least triplicate samples. Statistical significance was determined by Student's t-test or two-way ANOVA in GraphPad Prism 5.0. $P < 0.05$ was considered significant (indicated by an asterisk in the figures), $P < 0.01$ (indicated by two asterisks in the figures), $P < 0.001$ (indicated by three asterisks in the figures).

Supplementary References

1. Lam, Y.W. et al. Isolation of nucleoli. In Cell Biology: A Laboratory Handbook, Volume 2, Third Edition, J.E. Celis, ed. (Burlington, MA: Elsevier Academic Press), pp. 103–108 (2006).
2. Yin, Q. F. et al. SnoVectors for nuclear expression of RNA. Nucleic Acids Res 43, e5 (2015).
3. Kuhn, A. & Grummt, I. A novel promoter in the mouse rDNA spacer is active in vivo and in vitro. Embo J 6, 3487-92 (1987).
4. Gromava, J. E. Protein detection in gels by silver staining: a procedure compatible with mass-spectrometry. Cell Biology: A Laboratory Handbook. 3rd Edition. (2006)
5. Maamar, H., Cabili, M. N., Rinn, J. & Raj, A. linc-HOXA1 is a noncoding RNA that represses Hoxa1 transcription in cis. Genes Dev 27, 1260-71 (2013).
6. Schmidt, E. K., Clavarino, G., Ceppi, M. & Pierre, P. SUnSET, a nonradioactive method to monitor protein synthesis. Nat Methods 6, 275-7 (2009).
7. Liu, Q. et al. Amyloid precursor protein regulates brain apolipoprotein E and cholesterol metabolism through lipoprotein receptor LRP1. Neuron 56, 66-78 (2007).
8. Dong, Z. W. et al. RTL-P: a sensitive approach for detecting sites of 2'-O-methylation in RNA molecules. Nucleic Acids Res 40, e157 (2012).
9. Guo, P. et al. Rapid and simplified purification of recombinant adeno-associated virus. J Virol Methods 183, 139-46 (2012).
10. Vorhees, C. V. & Williams, M. T. Morris water maze: procedures for assessing spatial and related forms of learning and memory. Nat Protoc 1, 848-58 (2006).
11. Jain, S. et al. Arf4 determines dentate gyrus-mediated pattern separation by regulating dendritic spine development. PLoS One 7, e46340 (2012).
12. Lugo, J. N., Smith, G. D. & Holley, A. J. Trace fear conditioning in mice. J Vis Exp (2014).
13. Dunkley, P. R., Jarvie, P. E. & Robinson, P. J. A rapid Percoll gradient procedure for preparation of synaptosomes. Nat Protoc 3, 1718-28 (2008).
14. Zaqout, S. & Kaindl, A. M. Golgi-Cox Staining Step by Step. Front Neuroanat 10, 38 (2016).
15. Liu, Q. et al. Lipoprotein receptor LRP1 regulates leptin signaling and energy homeostasis in the adult central nervous system. PLoS Biol 9, e1000575 (2011).
